# Potential therapeutic targets for ischemic stroke in pre-clinical studies: Epigenetic-modifying enzymes DNMT/TET and HAT/HDAC

**DOI:** 10.3389/fphar.2025.1571276

**Published:** 2025-04-28

**Authors:** Yurou Guo, Jing Li, Xiaodan Liu, Huang Ding, Wei Zhang

**Affiliations:** ^1^ School of Integrated Chinese and Western Medicine, Hunan University of Chinese Medicine, Changsha, China; ^2^ Key Laboratory of Hunan Provincial for Integrated Traditional Chinese and Western Medicine on Prevention and Treatment of Cardio-Cerebral Diseases, Changsha, China

**Keywords:** epigenetics, ischemic stroke, DNMT, TET, HAT, HDAC, natural compounds

## Abstract

Ischemic stroke (IS) remains a leading cause of mortality and disability worldwide, driven by genetic predispositions and environmental interactions, with epigenetics playing a pivotal role in mediating these processes. Specific modifying enzymes that regulate epigenetic changes have emerged as promising targets for IS treatment. DNA methyltransferases (DNMTs), ten-eleven translocation (TET) dioxygenases, histone acetyltransferases (HATs), and histone deacetylases (HDACs) are central to epigenetic regulation. These enzymes maintain a dynamic balance between DNA methylation/demethylation and histone acetylation/deacetylation, which critically influences gene expression and neuronal survival in IS. This review is based on both *in vivo* and *in vitro* experimental studies, exploring the roles of DNMT/TET and HAT/HDAC in IS, evaluating their potential as therapeutic targets, and discussing the use of natural compounds as modulators of these enzymes to develop novel treatment strategies.

## 1 Introduction

According to recent reports, stroke is projected to cause nearly 10 million deaths by 2050 (The Lancet, 2024). Already a leading contributor to the global disease burden, stroke remains the second leading cause of death and the third leading cause of disability worldwide (GBD, 2024). IS, which account for 87% of all stroke cases (Martin et al., 2024), result from impaired cerebral blood circulation. This leads to ischemia and hypoxia, causing pathological damage, such as neuroglial cell injury, excitatory neurotoxicity, and mitochondrial dysfunction, which subsequently trigger a series of pathophysiological cascade responses. Current treatments for IS primarily focus on intravenous thrombolysis and mechanical thrombolysis. However, its short therapeutic window and high surgical risk underscore the urgent need to explore novel treatment approaches (Powers et al., 2019).

**FIGURE 1:**
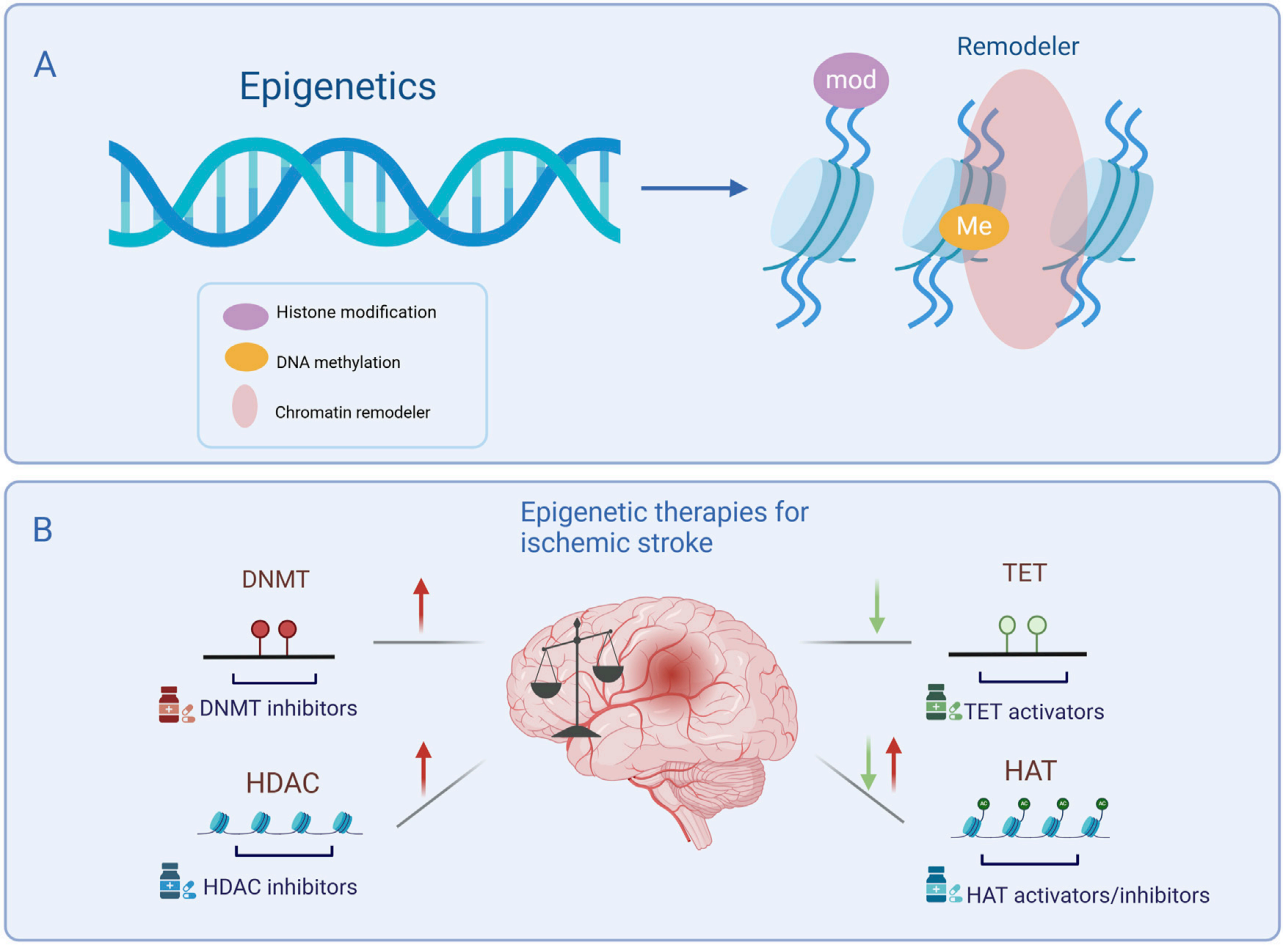
Main types of epigenetic modifications and targeting epigenetic enzymes in ischemic stroke treatment. **(A)** Epigenetic modifications include histone modification, DNA methylation, and chromatin remodeling. **(B)** Epigenetic therapies for ischemic stroke. Following ischemic stroke, DNMT and HDAC levels are generally elevated and can be suppressed by specific inhibitors, while TET levels are generally downregulated, requiring activators to restore function. HAT levels may be either upregulated or downregulated depending on specific pathological conditions, necessitating either inhibitors or activators to maintain epigenetic balance. Targeting these enzymes presents a potential therapeutic strategy for ischemic stroke. DNMT, DNA methyltransferase; TET, Ten-eleven translocation; HAT, Histone acetyltransferase; HDAC, Histone deacetylase. Symbols: Red arrows indicate the upregulation of epigenetic enzyme expression levels following ischemic stroke, while green arrows indicate their downregulation.

Epigenetics play a crucial role in the pathogenesis of central nervous system diseases (Bertogliat et al., 2020), with evidence demonstrating its involvement in the regulation of brain injury and neurological deficits (Aldridge and West, 2024; Singh et al., 2025). It is also essential for brain development and maintenance of brain function (LaSalle, 2025; Xu et al., 2024). Epigenetic modifications often occur through mechanisms such as DNA methylation, histone modification, and chromatin remodeling (Figure 1A). Enzymes responsible for these modifications are key regulators of gene expression. Thus, targeting epigenetic enzymes may provide new therapeutic targets for IS. This article focuses on the therapeutic potential of targeting two dynamically balanced enzyme systems in IS, DNMT/TET and HDAC/HAT (Figure 1B).

## 2 DNA methylation

DNA methylation plays a crucial role in epigenetic reprogramming and cellular function. Cytosine methylation at the 5th position is one of the most well-understood epigenetic modifications (Smith and Meissner, 2013). DNA methylation, regulated by DNMTs, involves the transfer of a methyl group from donors such as S-adenosylmethionine to the cytosine residue in CpG sites on the gene, resulting in 5-methylcytosine (5mC). This modification condenses the chromatin structure and suppresses transcription and gene expression. DNMT deficiency can severely disrupt normal development, often leading to early embryonic lethality (Okano et al., 1999). Maintaining a hypomethylated state in promoter CpG islands requires active involvement of DNMTs (Smith and Meissner, 2013). In mammals, three main DNMTs mediate the methylation process: DNA methyltransferase 1 (DNMT1), DNA methyltransferase 3A (DNMT3A), and DNA methyltransferase 3 B (DNMT3B).

**FIGURE 2:**
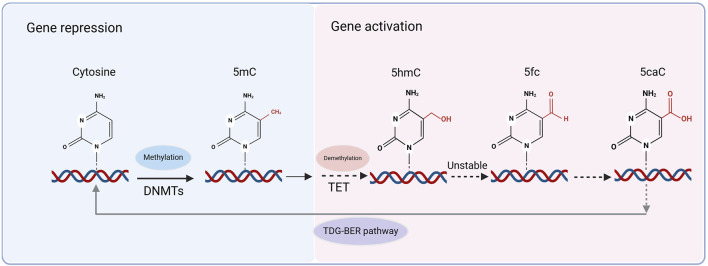
Methylation and demethylation processes. DNA methylation is catalyzed by DNA methyltransferases (DNMTs), converting cytosine to 5-methylcytosine (5mC), which is associated with gene repression. Active DNA demethylation occurs through the ten-eleven translocation (TET) enzymes, which sequentially oxidize 5mC to 5-hydroxymethylcytosine (5hmC), 5-formylcytosine (5fC), and 5-carboxylcytosine (5caC). Due to the relative instability of 5fC and 5caC, these modified cytosines are recognized and excised by thymine DNA glycosylase (TDG), initiating the TDG-mediated base excision repair (TDG-BER) pathway, which restores unmethylated cytosine and completes the demethylation process.

Mammalian DNA demethylation is mediated by the TET protein family. TET enzymes catalyze the addition of a hydroxyl group to 5-mC to form 5-hydroxymethylcytosine (5hmC), which is considered the first step in DNA demethylation (Yamaguchi et al., 2013). Further oxidation of 5hmC resulted in the formation of 5-formylcytosine (5fC) and 5-carboxylcytosine (5caC) (Lu et al., 2014). Owing to the relative instability of 5fC and 5caC in chromatin, they are recognized and removed by thymine-DNA glycosylase (TDG). Subsequently, through the TDG-mediated base excision repair (BER) pathway, a cascade of repair enzymes restores the unmethylated cytosine, completing the active demethylation process (Figure 2). This provides compelling evidence for DNA demethylation (He et al., 2011). 5hmC acts as an intermediate in DNA demethylation and 5hmC-mediated active demethylation is required for mammalian neuronal differentiation and function (Stoyanova et al., 2021). The TET family of enzymes involved in this process includes three subtypes: TET1, TET2, and TET3, all of which are widely expressed in the brain (Choi et al., 2022). DNA methylation is a dynamic process that is primarily maintained through the cooperation of DNMTs and TET proteins.

### 2.1 DNA methyltransferases and their functions

#### 2.1.1 DNMT1

DNMT1 is responsible for maintaining the methylation status of the genome (Berkyurek et al., 2014; Chialastri et al., 2024). DNMT1 consists of a C-terminal structural domain and a large portion of an N-terminal structural domain that interact with each other in order to catalyze the enzyme activity, which in turn regulates the DNA methyltransferase function of the variant (Fatemi et al., 2001). DNMT1 is an indispensable methyltransferase during embryonic development, playing a key role in chromatin structure (Martino et al., 2024), neuronal survival (Bayer et al., 2020), and cell cycle regulation (Yang et al., 2024). The absence of DNMT1 leads to rapid cell death in human embryonic stem cells (Liao et al., 2015) and causes lethal damage to dividing somatic cells (Trowbridge et al., 2009). During the mitotic S phase, DNMT1 inhibits the Checkpoint Kinase 1 (Chk1) pathway to protect the differentiated neurons (Oshikawa et al., 2017). Studies have shown that DNMT1 is crucial for the development of the dentate gyrus in mice, and its absence leads to abnormalities in the molecular and granule cell layers of the dentate gyrus (Noguchi et al., 2016). Mutations in DNMT1 in the dorsal forebrain of mice result in significant cortical and hippocampal degeneration accompanied by neurobehavioral deficits in learning and memory (Hutnick et al., 2009). DNMT1 expression is also age-dependent, with high expression levels in the cortex and hippocampus of young mice, which gradually decrease with age (Singh and Thakur, 2014).

#### 2.1.2 DNMT3

The DNMT3 family includes DNMT3A, DNMT3B, and DNMT3L, with DNMT3A and DNMT3B referred to as *de novo* methyltransferases (Okano et al., 1999), in early embryonic development, DNMT3A and DNMT3B are co-expressed in the epiblast. Their inactivation blocks *de novo* methylation in early embryos, but does not affect the maintenance of genomic DNA methylation, which is associated with the establishment of new methylation patterns on previously unmodified DNA during development (Monsour et al., 2022) DNMT3A has a lower enzymatic activity than DNMT1, which may be related to its need for specific interactions with the cofactor ubiquitin like with PHD and ring finger domains 1 (Uhrf1) during the catalytic process. DNMT3 is involved in the formation of long-term memory in hippocampal neurons activated by memory events (Bernstein, 2022). Another regulatory factor, DNMT3L, stimulates the activity of DNMT3A and DNMT3B in *de novo* DNA methylation (Ooi et al., 2007), acting as an auxiliary protein for DNMT3A and DNMT3B, and participating in the regulation of repetitive regions and imprinting in germ cells (Bourc’his et al., 2001). The DNMT3L knockout did not affect the development of normal mice. Increasing evidence suggests that DNMT3A and DNMT3B contribute to the maintenance of methylation during replication, and *de novo* methylation may also require the involvement of DNMT1 (Egger et al., 2006), indicating that the process of methylation is not the action of a single methyltransferase but requires cooperation among each other.

#### 2.1.3 DNA methyltransferases in ischemic stroke

The involvement of DNMTs in ischemic stroke pathogenesis is gaining increasing recognition (Stanzione et al., 2020). Cerebral ischemic injury leads to alterations in gene expression, which are crucial for the repair of cellular damage in the ischemic region (Choi et al., 2022). After cerebral ischemia, overall DNA methylation levels in the brain are upregulated, inhibiting gene transcription and expression (Endres et al., 2000). This inhibition exacerbates brain damage and correlates with increased DNMT activity in the brain. Consequently, DNA methylation may contribute to secondary brain damage following stroke, and inhibition of DNA methylation may enhance resistance to ischemia (Endres et al., 2001).

##### 2.1.3.1 *In vitro* studies

DNMT1 plays a crucial role in regulating synaptic functions of excitatory and inhibitory interneurons (Pensold et al., 2020). An *in vitro* study showed that inhibition of DNMT1 expression impaired the proliferation of HT22 neuronal cells derived from the mouse hippocampus and exacerbated apoptosis, thereby affecting brain function (Yang J. et al., 2017). DNMT1 was also associated with microglial polarization after stroke. A study showed that DNMT1 was upregulated in activated microglia, and silencing DNMT1 promoted M2 polarization of microglia, thereby reducing neuroinflammation (Tan et al., 2022). DNMT1 binds to other proteins to form complexes that exert neuroprotective effects. For example, Repressor-Element 1 (RE1)-silencing transcription factor (REST) downregulated Na^+^/Ca^2+^exchanger-1 (Ncx1) in stroke by forming a complex with the epigenetic writer DNMT1 and the epigenetic reader methyl-CpG binding protein 2 (MeCP2). Using primary mouse cortical neurons, oxygen-glucose deprivation/reperfusion (OGD/R) for 24 h, REST, DNMT1, and MeCP2 expression increased, while Ncx1 protein expression decreased at 12 and 24 h. Silencing of DNMT1, MeCP2, and REST, as well as inhibition of DNA methylation, significantly prevented OGD/R-induced downregulation of Ncx1 and prevented neuronal death (Guida et al., 2024). DNMT3A is involved in the regulation of mitochondrial autophagy following cerebral ischemia-reperfusion injury. The overexpression of DNMT3A leads to increased neuronal apoptosis after OGD/R injury, diminishing its protective effects on neurons (Zhu et al., 2024).

##### 2.1.3.2 *In vivo* studies

DNMT mediates DNA methylation. Ischemia and reperfusion may cause oxidative damage to DNA and DNMT1 has been shown to stimulate DNA repair (Sharifulina et al., 2021). *In vivo* studies have shown that after transient middle cerebral artery occlusion/reperfusion (MCAO/R), DNMT levels in ischemic brain tissue increase in the striatum and cortex (Endres et al., 2000), with a significant increase in 5-mC levels in the cerebral cortex (Asada et al., 2020), which may contribute to cell death (Chestnut et al., 2011). Reducing DNMT1 expression and 5-mC levels, along with hypomethylation of synaptic gene DNA, has been found to protect synaptic function in the hippocampus of rats (Shi et al., 2023). The reduction in DNMT1 expression may be related to delayed neuronal death caused by transient cerebral ischemia (Lee et al., 2013). Although reducing DNMT1 levels can protect mice from the effects of cerebral ischemia, the absence of DNMT1 does not prevent ischemic brain injury (Endres et al., 2001). Interestingly, mice with a double knockout of DNMT1 and DNMT3A exhibited abnormal synaptic plasticity in the hippocampal CA1 region and deficits in learning and memory (Feng et al., 2010). However, an opposite trend was observed in diabetic stroke models. After ischemic injury, DNMT1, DNMT3A, and overall 5-mC levels decreased in the brains of diabetic mice, whereas these levels increased in nondiabetic brains following ischemic insult (Kalani et al., 2015).

Junhe Cui et al. found that targeting DNMT3B after focal ischemia in rats reduced long interspersed nuclear element-1 (LINE-1) methylation levels (Cui et al., 2020) and inhibiting DNMT3B-mediated methylation can reduce neuronal apoptosis and improve neurological function in IS (Deng et al., 2020). However, this contrasts with previous reports suggesting that inhibiting DNMT function exerted neuroprotective effects, where inhibition of DNMT3A in a mouse model of transient cerebral ischemia increased infarct volume and exacerbated neurobehavioral impairments, with an increase in the number of neutrophils and infiltration of peripheral blood and central neutrophils (Lyu et al., 2024). DNMT can be targeted directly or indirectly to treat stroke. Elevated homocysteine (Hcy) level is a risk factor for IS. Hcy inhibits the proliferation of neural stem cells by decreasing DNMT activity and overall methylation levels in the hippocampus of the brain tissue of middle cerebral artery occlusion (MCAO) rats. Hcy-induced DNA hypomethylation may be caused mainly by a decrease in DNMT activity. Maintaining normal DNA methylation levels by lowering Hcy levels provides a new therapeutic approach to promote neurological recovery and reconstruction after stroke (Gou et al., 2021).

### 2.2 DNA demethylases and their functions

#### 2.2.1 TET1

TET-mediated DNA demethylation occurs actively in neuronal enhancers and promotes cell identity (Tian et al., 2024). The TET1 protein is notably expressed in the fetal brain, heart, spleen, and kidney, with additional expression observed in adult ovaries and skeletal muscle (Lorsbach et al., 2003). TET1 catalyzes a significant increase in 5hmC levels, where hydroxymethylation modifications are essential for genomic function. Interestingly, the transcription start sites (TSS) of gene promoters bound by TET1 are typically devoid of DNA methylation, in contrast to TET1-unbound promoters, which are often methylated (Wu et al., 2011). The depletion of TET1 leads to a reduction in 5hmC levels at the TSS (Huang et al., 2014). Although the deletion of TET1 does not substantially impact the demethylation process (Yamaguchi et al., 2012), the downregulation or knockout of TET1 results in decreased 5hmC levels and significant transcriptional alterations (Williams et al., 2011). Mice lacking TET1 exhibit neurological changes in the hippocampus, including deficits in learning and memory (Zhang et al., 2013), suggesting a role for TET1 in memory formation. In embryos with a double knockout of TET1 and TET3, a decrease in 5hmC and increase in 5mC have been observed (Liu et al., 2019).

#### 2.2.2 TET2

TET2 is prominently expressed in various organs including the heart, liver, spleen, and lungs (Lorsbach et al., 2003). This enzyme plays a pivotal role in regulating the balance between 5hmC and 5mC, which is essential for preserving genomic integrity (Joshi et al., 2022). The absence of TET2 results in diminished hydroxymethylation and a concurrent increase in DNA methylation levels, particularly in the enhancer regions (Hon et al., 2014). Intriguingly, the expression levels of TET2 in mouse embryonic stem cells aligned with the detectable presence of 5hmC, 5fC, and 5caC (Ito et al., 2010). Loss of neuronal Tet2 enhances hippocampal-dependent cognitive function. TET2 deletion in young adult mice impairs neurogenesis, whereas TET2 overexpression suppresses hippocampus-dependent memory. However, in the hippocampus of aged mice, decreased TET2 expression and 5hmC levels correlate with a decline in neurogenesis (Pratt et al., 2023).

#### 2.2.3 TET3

TET3 is highly expressed in the brain, where 5hmC is the most abundant (Antunes et al., 2021). It plays a crucial role in synaptic gene regulation by modulating 5hmC levels in neurons (Liu et al., 2024). TET3 is associated with biological processes, such as synaptic transmission (Yu et al., 2015), and its dysregulation disrupts homeostatic synaptic plasticity. Additionally, TET3 serves as a key neuroprotective factor by regulating lysosomal and autophagy-related genes, thereby preventing neuronal death (Jin et al., 2016). Deletion of TET3 leads to abnormal differentiation of neural stem cells, manifested by downregulation of neural gene expression (MacArthur et al., 2024).

#### 2.2.4 DNA demethylases in ischemic stroke

##### 2.2.4.1 *In vitro* studies

In an *in vitro* study, TET1 knockdown led to downregulation of genes that regulate neuronal activity (Santiago et al., 2020). It also promoted oxidative stress, which in turn induced neuronal apoptosis (Xin et al., 2015). Conversely, TET1 overexpression in astrocytes protected against ischemia-induced autophagy and apoptosis, induced promoter hypomethylation, and potentially mitigated brain damage caused by IS (Zhou D. et al., 2021). A recent study indicated that overexpression of TET1 in microglia increased the M1/M2 polarization ratio, exacerbating neuroinflammation induced by cerebral ischemia/reperfusion (Lin Y. et al., 2024). Therefore, the expression of TET enzymes should be maintained at an optimal dynamic equilibrium, neither excessively high or low, to ensure proper physiological function and prevent adverse effects. Using BV2 microglia, Qingyi Ma et al. found that knocking down TET2 worsened neonatal HI-induced brain infarct and neurological deficits and reversed the neuroprotective effect of miR-210 inhibition, the miR-210-TET2 axis regulated pro-inflammatory response in microglia (Ma et al., 2021).

##### 2.2.4.2 *In vivo* studies

A growing body of evidence implicates TET enzymes and 5hmC in neuroprotection, with increased 5hmC levels being associated with enhanced neurological recovery following cerebral ischemia (Bertogliat et al., 2020). 5hmC was rapidly elevated after cerebral ischemia/reperfusion injury (CI/RI) in mice and remained elevated for 48h, TET2 expression was increased, and TET2 protein knockdown increased the area of cerebral infarction after MCAO (Miao et al., 2015). Furthermore, it has been observed that TET3 levels rise in peri-infarct cortical neurons and astrocytes of mice within 24 h of focal ischemia onset. By contrast, the expression levels of TET1 and TET2 remain stable during this period. Notably, the increase in 5hmC levels post-ischemia is TET3-dependent; suppression of TET3 not only reduces 5hmC levels, but also exacerbates ischemic brain injury. These findings suggest that TET3 plays a role in endogenous neuroprotection (Morris-Blanco et al., 2021). Elevated 5hmC levels may be mediated by TET3 or Tet2 in the peri-infarct region, favoring neurological recovery after cerebral ischemia (Bertogliat et al., 2020). In mice, the abundance of 5hmC in mitochondrial DNA increases after ischemic brain injury, and inhibition of Tet2 decreases 5hmC expression while increasing cellular ATP levels (Ji et al., 2018), leading to mitochondrial dysfunction, which in turn exacerbates brain injury. Furthermore, a comprehensive decrease in cortical 5hmC has been documented following ischemic-hypoxic brain injury in rats, potentially related to the downregulation of TET1 and TET2 (Zhang et al., 2019b). Using a mouse model of poststroke depression (PSD) induced by MCAO and spatial constraint stress, researchers found that decreased in TET2 expression in the brain caused PSD by decreasing Wnt/β-catenin/Lymphoid Enhancer Factor-1 (Wnt/β-catenin/LEF1) pathway signaling to promoting inflammatory factor IL-18 expression (Wei et al., 2021).

## 3 Histone modification

**FIGURE 3:**
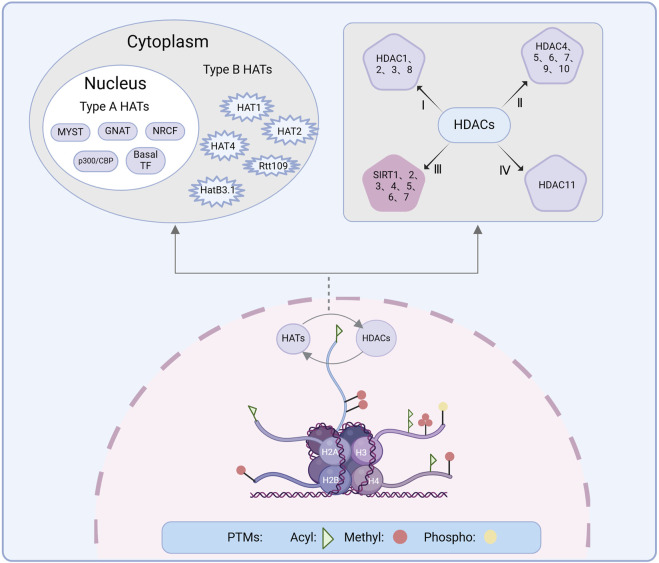
Histone modification is the PTM of histone amino-terminal tails under the action of relevant enzymes, producing modification processes such as acetylation, phosphorylation, methylation, among others of which acetylation is dynamically regulated by HATs and HDACs. The upper left shows the classification of HATs. HATs are categorized into two types: Type A HATs include the GNAT family, the p300/CBP family, the MYST family, the basal TF family, and the NRCF family. Type B HATs include HAT1, HAT2, HatB3.1, Rtt109, and HAT4. The upper right shows the classification of HDACs, which is categorized into four types. PTM, Post-translational modification; HAT, Histone acetyltransferase; HDAC, Histone deacetylase; Acyl, Acetylation; Methyl, Methylation; Phospho, Phosphorylation.

Histone modification involves post-translational modifications (PTMs) on the amino-terminal tails of histones, catalyzed by specific enzymes, including acetylation, phosphorylation, and methylation (Zhao et al., 2024) (Figure 3). Among these modifications, histone acetylation has proven to be more effective for chromatin dynamics and gene expression. Consequently, acetylation has been particularly well studied in the context of neurological disorders (Bhaumik et al., 2007). Histone acetylation facilitated by HATs involves the addition of acetyl groups to specific lysine residues on the N-terminal tails of core histones, thereby promoting transcriptional activation in eukaryotes (Hebbes et al., 1988). However, this process is reversible: HDACs counteract HATs by removing acetyl groups and inhibiting gene expression through the deacetylation of ε-amino acids on conserved lysine residues within the N-terminal tails of histones (Xia et al., 2007). The interplay between HATs and HDACs, two enzymes with opposing functions, dynamically regulates gene expression. This delicate balance is crucial not only for gene expression but also for DNA repair and modulation of disease states (Haberland et al., 2009).

### 3.1 Histone acetyltransferases

HATs are primarily classified into two types: type A (nuclear) and type B (cytoplasmic). Type A HATs are involved in histone acetylation within chromatin to promote transcription, whereas type B HATs acetylate newly synthesized histones to influence the nucleosome structure (Qiu et al., 2025). HATs mediate many different biological processes, such as gene regulation and DNA damage repair (Marmorstein and Zhou, 2014), and play a key role in both the developing and adult brain (Kabir et al., 2022). CREB-binding protein (CBP)/p300 and p300/CBP-associated factors (P/CAFs) are key players in neuronal growth and development. Neuronal activity promotes repetitive CREB-CRE and CREB-CBP interactions, leading to rapid gene expression (Atsumi et al., 2024). CBP/p300 also regulates the expression of brain-derived neurotrophic factor in rats, in collaboration with the cAMP-response element binding protein (CREB) family of transcription factors, which are essential for neuron survival, synaptic plasticity, and other functions (Esvald et al., 2020). Mice with mutations in the CBP/p300 genes exhibit pronounced deficits in learning and memory (Goodman and Smolik, 2000), and the deletion of CBP and p300 leads to the failure of neural tube closure and extensive cerebral hemorrhage (Tanaka et al., 2000). Furthermore, the absence of these proteins induces the abnormal migration of motor neuron cell bodies, accompanied by a significant reduction in histone H3 acetylation in motor neuron enhancers (Lee et al., 2009). A previous study also suggested that p300 may be involved in oligodendrocyte differentiation in the developing rat brain (Zhang et al., 2016). Tip60 was the first HAT found *in vitro* in primary hippocampal neurons to undergo nucleoplasmic translocation in neurons (Karnay et al., 2019), which is critical for regulating plasticity genes in response to the environment (Armour et al., 2023). Disruption of Tip60 HAT-mediated neural histone acetylation homeostasis is a common early event in neurological disorders in a previous report (Beaver et al., 2020).

#### 3.1.1 Histone acetyltransferases in ischemic stroke

HAT1 is thought to be a cytoplasmic protein involved in the regulation of protein expression profiles after IS (Ortega et al., 2023). S V Demyanenko et al. constructed a photothrombotic stroke (PTS) model using rats, and they found that the expression of HAT1 and P300/CBP-associated factor (PCAF) was upregulated in the ischemic semi-dark band of the rat cerebral cortex at 4–24 h after PTS, and that HAT1 being localized in astrocytes (Demyanenko and Uzdensky, 2019). To further understand the localization and expression of HAT1 and PCAF in the core infarct zone induced by stroke, they conducted additional experiments and found that in the PTS model, PCAF levels remained unchanged in the nuclear fraction, whereas HAT1 was overexpressed only at 24 h after PTS. However, both HAT1 and PCAF were upregulated in the cytoplasm, which may be mainly related to their overexpression in the cytoplasm of neurons, especially astrocytes (Demyanenko et al., 2020c). Astrocytes respond rapidly after ischemia, and HAT1 may be involved in the process of astrocyte response in the injured brain. The level of p53 acetylation was significantly increased after the induction of a global cerebral ischemia model after bilateral ovariectomy in SD female rats. CBP/p300, an acetylase that targets and inhibits p53, has been shown to exert strong neuroprotective effects on the hippocampal CA1 region after overall cerebral ischemia, thereby reducing neuronal cell apoptosis (Raz et al., 2011).

HDAC3 cooperates with p300 to regulate oligodendrocyte and astrocyte phenotypes, and HDAC3 interacts with p300 to maintain oligodendrocyte identity while inhibiting signal transducer and activator of transcription 3 (Stat3)-mediated astrocytogenesis (Zhang et al., 2016). In addition, p300 is involved in activating ncx1 transcription. Oxygen and glucose deprivation plus reoxygenation (OGD/RX) treatment of cortical neurons has revealed that inhibition of p300 leads to increased neuronal damage and prevents the increase in ncx1 in cortical neurons. By contrast, upregulation of p300 activity enhances cellular resistance to ischemia and reduces the post-ischemic damage (Formisano et al., 2015). Enhanced activity of matrix metalloproteinase-9 (MMP-9) leads to disruption of the blood-brain barrier (BBB), neuroinflammation, and neuronal apoptosis. Increased MMP-9 promoter activity requires HAT activity of PCAF and CBP/p300 co-activators, and knockdown of these co-activators decreases MMP-9 expression (Zhao and Benveniste, 2008). Although changes in the activity of HATs may be associated with neuroprotective mechanisms, research on HATs in stroke remains relatively limited, highlighting an avenue for future investigation.

### 3.2 Histone deacetylases

HDACs can be divided into four classes based on sequence similarity and structure: Class I (HDAC1-3 and 8), Class II (HDAC4-7, 9, and 10), and Class IV (HDAC11), all of which are zinc-dependent enzymes. Class II is further divided into Class IIa (HDAC4, 5, 7, and 9) and IIb (HDAC6 and 10). Class III HDACs, also known as sirtuins (SIRT), are nicotinamide adenine dinucleotide (NAD)-dependent (Milazzo et al., 2020). HDACs exhibit distinct subcellular distributions *in vivo*. Class I HDACs are predominantly nuclear, while Class II and IV HDACs can shuttle between the nucleus and cytoplasm (Johnstone, 2002). The catalytic region of Class I HDACs contains conserved tyrosine residues, which are replaced by histidines in Class IIa HDACs (Park and Kim, 2020). Class IIa HDACs possess a unique feature: an extended N-terminal regulatory domain in addition to the core HDAC structure. Unlike Class I and IIb HDACs, Class IIa HDACs are not active on acetylated substrates (Lahm et al., 2007). Instead, Class IIa members recruit various protein modifications to inactivate transcription factors and enforce transcriptional repression (Guttzeit and Backs, 2022). HDAC11, the sole member of Class IV HDACs identified to date (Liu et al., 2020), is widely distributed in tissues such as the brain, heart, kidney, and skeletal muscle. The structure of HDAC11 remains elusive; however, structural modeling based on HDAC8 suggests that HDAC11 shares a structure similar to that of HDAC8 (Thangapandian et al., 2012). HDACs are not limited to deacetylation. They also participate in other PTMs that collectively regulate gene transcription and play crucial roles in modulating gene expression and metabolic processes (Zhang et al., 2024). HDACs play a crucial role in maintaining brain homeostasis (Rathore et al., 2021) by interacting with a variety of protein molecules and regulating numerous pathways (Cavalieri, 2021), including apoptotic cascades (Lu et al., 2023), inflammatory mechanisms (Dai et al., 2021), and protection of the BBB (Han et al., 2023), thus exerting neuroprotective effects.

Seven SIRTs (SIRT1–SIRT7) have been identified in mammals. These proteins share a conserved NAD+-binding catalytic domain, and the distinct localisation and functions of various SIRT types depend on their sequence and length within the N-terminal and C-terminal structural domains (Wu et al., 2022). SIRT1 is expressed in the brain, liver, muscle, endothelium, pancreas, and adipose tissue (Carrizzo et al., 2022), and is predominantly distributed in neurons of the cortex, hippocampus, cerebellum, and thalamus (Ramadori et al., 2008), primarily located in the nucleus and shuttling to the cytoplasm (Tanno et al., 2007). SIRT1 deacetylates histone H4 lysine 16 (H4K16), H3K9, and H1K26, leading to transcriptional repression (Zhang et al., 2019a). SIRT2 is mainly found in the cytoplasm, translocates to the nucleus during the G2/M phase of the cell cycle (Vaquero et al., 2006), and is able to maintain brain glucose homeostasis (Park et al., 2015). SIRT3, SIRT4, and SIRT5 are primarily located in the mitochondria (Satoh and Imai, 2014). SIRT3 activation in response to oxidative stress regulates mitochondrial proteins and inhibits the production of mitochondrial reactive oxygen species (ROS) (Tao et al., 2010). The absence of SIRT3 exacerbates mitochondrial autophagy and disrupts mitochondrial homeostasis (Li et al., 2018). SIRTs catalyze acetylation, and their high expression in the brain and multiple targets alters multiple biological processes in response to ischemic stimuli, including attenuating inflammatory responses, inhibiting oxidative stress, modulating autophagy, protecting the BBB integrity, and promoting angiogenesis (Liu et al., 2023; Wu et al., 2022).

#### 3.2.1 Histone deacetylases in ischemic stroke

##### 3.2.1.1 *In vitro* studies

An *in vitro* experiment showed that HDAC1 mediated the transformation of microglial phenotypes after stroke. Overexpression of HDAC1 reduced the acetylation of histones H3 and H4, significantly decreasing microglial viability in an oxygen-glucose deprivation (OGD) model, whereas downregulation of HDAC1 promoted the transformation of M1-type microglia to the M2 phenotype (Wang et al., 2017). HDAC1 knockout reduced the expression of inflammatory factors in lipopolysaccharide (LPS)-induced BV2 microglia *in vitro*, suggesting that HDAC1 inhibitors may play a significant role in targeting brain damage and neuroinflammation (Durham et al., 2017). Similarly, HDAC3 acted as a hub to induce microglial phenotypic transformation. For example, HDAC3 activation exacerbated neuroinflammation, whereas HDAC3 deficiency promoted the development of an anti-inflammatory microglial phenotype (Zhao et al., 2022). Expression of HDAC3 was increased in microglia after stroke. HDAC3 activated the transcription factor PU.1 through chromatin remodeling, and this activation further promoted the proliferation of pro-inflammatory microglia (Inglis, 2024).

SIRT3 played a protective role in IS. A study showed that upregulating of SIRT3 through the SIRT3-FOXO3a-SOD2 pathway reduced brain ischemia-reperfusion injury and repaired neurons (Yin et al., 2015). Astroglial activation formed neuroglial scars post-stroke, and Foxo3a was an important mediator of the inhibition of astroglial proliferation (Cui et al., 2011). Knockdown of SIRT3 weakened the inhibitory effect of drugs on astroglial proliferation, whereas overexpression of SIRT3 activated the inhibition of astroglial activation through the SIRT3-FOXO3a pathway, reducing neuroglial scar formation and promoting angiogenesis after IS (Yang X. et al., 2017). Furthermore, overexpression of SIRT3 activated the mitochondrial unfolded protein response (Xiaowei et al., 2023), maintained mitochondrial homeostasis after CI/RI, and restored mitochondrial structure and function (Chen H. et al., 2024). In SIRT3 knockout mice post-stroke, neuroprotection occurred through a compensatory increase in SIRT1 levels, independent of the loss of SIRT3 (Verma et al., 2019). SIRT7 also exerted neuroprotective effects, and overexpression of SIRT7 regulated the p53-mediated pro-apoptotic signaling pathway in the OGD/R model, protected neurons by inhibiting apoptosis (Lv et al., 2017).

The role of SIRT5 in IS remained controversial. SIRT5 maintained mitochondrial energy metabolism and protected against metabolic stressors in the brain by offered mitochondrial protection after ischemic injury (Morris-Blanco et al., 2016). Overexpression of SIRT5 under OGD/R conditions exacerbated microglia-induced neuroinflammation and increased neurological deficits. Conversely, SIRT5 knockdown prevented microglial overactivation and reduced pro-inflammatory factor expression, infarct volume, and neurological deficits (Xia et al., 2022). Nevertheless, recent research suggested that blocking SIRT5 enhanced glutamine metabolism and excitotoxicity, thereby worsening brain ischemic injury (Wang J. et al., 2024).

Two studies demonstrated the neuroprotective effects of SIRT6 in ameliorating neuroinflammation (He et al., 2021) and attenuating neuronal death (Cheng et al., 2021). Overexpression of SIRT6 attenuated OGD-induced neuronal death (Cheng et al., 2021) and activated mitochondrial autophagy in IS (Song et al., 2024). Inhibition of Notch signaling ameliorated cellular damage and promoted angiogenesis in OGD/R-induced neuronal cells (Xiao et al., 2023). In hypoxia/reoxygenation-induced primary human brain microvascular endothelial cells, SIRT6 silencing impaired cellular barrier function and reduced cell viability. Endothelial SIRT6 played a protective role in IS by maintaining BBB integrity (Liberale et al., 2020).

##### 3.2.1.2 *In vivo* studies

Different HDACs had distinct roles in IS. Class I HDACs were widely distributed throughout the brain (Baltan et al., 2011). HDAC1 regulated neuronal viability and was neuroprotective in mice after IS. HDAC1 dysfunction exacerbated neuroinflammation and BBB destruction after cerebral ischemia-reperfusion injury (Wang et al., 2023). However, a study with contrasting results suggested that HDAC1 had cytotoxic effects, promoted apoptosis and impaired mitochondrial transport; only inhibition of HDAC1 improved mitochondrial transport (Kim et al., 2010). Ischemia-induced HDAC3 neurotoxicity was confirmed (Xia et al., 2007) and the neurotoxicity of HDAC3 was inseparable from its interaction with HDAC1. HDAC3 knockdown inhibited HDAC1-induced neurotoxicity and HDAC1 knockout inhibited HDAC3-induced neurotoxicity (Bardai et al., 2012).

The current understanding attributes post-stroke functional recovery to brain remodeling and neuroplasticity. Targeting HDAC2 has been considered a novel approach for promoting neurofunctional recovery. Six days after thrombotic stroke in mice, increased expression and activity of HDAC2 were detected in the peri-infarct cortex, accompanied by loss of motor function (Lin et al., 2017). HDAC2 overexpression inhibited synaptic remodeling, reduced dendritic spine density and synapse number, and led to memory decline (Guan et al., 2009). Conversely, HDAC2 deletion reversed the downregulation of synaptic proteins and parvalbumin induced by stroke, thereby promoting functional recovery in the brain (Tang Y. et al., 2017). HDAC2 may be a key mediator of post-stroke motor dysfunction.

HDAC4 was highly expressed in the brain, and stroke induced nuclear shuttling of HDAC4 in neurons of the peri-infarct cortex, which did not trigger neuronal cell death, but was positively correlated with neuronal repair after ischemic injury (Kassis et al., 2015). However, HDAC4 translocated from the cytoplasm to the nucleus during neuronal stress. This nuclear translocation induced OGD neuronal death and exacerbated the infarct area and functional deficits in MCAO mice, thereby worsening the outcome of stroke (Yuan et al., 2016). Downregulation of HDAC4 enhanced neuroprotection (Chen et al., 2025; Song et al., 2022). The expression and activity of HDAC5 significantly increased and the expression of histone acetyltransferase p300 decreased 6h after CI/RI in the rat brain. Overexpression of HDAC5 suppressed the anti-apoptotic effect of myosin-related transcription factor-A (MRTF-A) in the center of the cell, whereas overexpression of p300 enhanced MRTF-A-induced anti-apoptotic effects (Li et al., 2017). This also provided strong evidence for a dynamic balance between HATs and HDACs.

HDAC6 was expressed in the cortex and cerebellum of mice, and the transcription of HDAC6 was significantly upregulated in the penumbral region of the cortex after PTS-induced. The inhibition of HDAC6 played a neuroprotective role in ischemic brain injury (Hu et al., 2022). In the early stages following induced transient middle cerebral artery occlusion in mice, HDAC6 expression levels significantly increased, suggesting its potential as a mediator of neurotoxicity in IS (Chen et al., 2012). A recent study used positron emission tomography (PET) imaging to observe dynamic changes in HDAC6 in a mouse model of IS to assess its neuroprotective effects. These results suggested that the pharmacological inhibition of HDAC6 had significant anti-neuroinflammatory and neuroprotective effects (Zhou et al., 2024). Furthermore, HDAC6 was significantly overexpressed in neurons and astrocytes in the semi-dark band of the rat cerebral cortex induced by PTS, and additionally co-localized with TUNEL-positive apoptotic cells, HDAC6 may be involved in ischemia-induced apoptotic cell death (Demyanenko et al., 2020b).

HDAC8 expression was consistently increased in cortical neurons and astrocytes 3–14 days after photothrombotic infarction-induced mice, and this upregulation might have been associated with the recovery process after ischemia (Demyanenko et al., 2018). A recent study shed light on HDAC8’s role in mediating a reversible acetylation process, which served as a regulator of the cell cycle. This study indicated that glucose deprivation triggered the acetylation of HDAC8 at lysine 202, potentially leading to cell cycle arrest (Sang et al., 2024). Cell cycle arrest may exacerbate ischemic injury by affecting the normal function of neurons and glial cells and even leading to cell death.

HDAC9 played a crucial role in activity-dependent gene expression and dendritic growth of developing cortical neurons (Sugo et al., 2010) and was linked to the large vessel variations observed in IS (Brookes et al., 2018). HDAC9 expression was upregulated after cerebral ischemic injury (Zhang et al., 2023; Zhong et al., 2020). A previous study reported significant upregulation of HDAC9 expression in the ischemic hemisphere of rat brains following cerebral ischemia-reperfusion injury. This increase was associated with abnormal endothelial cell permeability, while the suppression of HDAC9 gene expression alleviated ischemia-induced endothelial damage and BBB disruption (Shi et al., 2016). Specific knockdown of HDAC9 from neurons in the peri-infarct region of MCAO mice reduced infarct volume, inhibited neuronal apoptosis, and improved neurological outcomes (Lin H. et al., 2024). Thus, HDAC9 inhibition could be a potential treatment for stroke (Markus, 2023).

SIRT1 plays a protective role in IS. Increased levels of SIRT1 during brain repair after stroke were associated with an increase in synaptic plasticity proteins, one of the key mechanisms by which the brain recovers its function after injury (Demyanenko S. et al., 2020). Research indicated that the Sirt1/Foxo3a axis might have served as a potential therapeutic pathway for MCAO/R (Tan et al., 2024), with activated SIRT1 reducing infarct volume in mouse brains, whereas SIRT1 knockout increased infarct volume (Hernández-Jiménez et al., 2013). A study that utilized nanotechnology for Sirt1-targeted nasal administration observed a significant reduction in brain edema caused by IS (Ryu et al., 2024). This also provided new evidence of the neuroprotective effects of Sirt1. SIRT2 was associated with stroke-induced neuroinflammation, microglial activation (Wu et al., 2020), and neuronal death (Zhang et al., 2021). Increased levels of SIRT2 were associated with α-microtubule protein acetylation, which decreased synapse mobility and adversely affected nerve recovery (Demyanenko S. et al., 2020). SIRT2 diminished the anti-inflammatory effects of infiltrating regulatory T cells (Treg cells) in MCAO mouse models and inhibited hypoxia-inducible factor 1-α (HIF-1α) expression in Treg cells, thereby eliminating the upregulation of SIRT2 (Shu et al., 2019). SIRT2 was essential for microglial activation (Wang B. et al., 2016). A study showed that inhibiting SIRT2 promoted the polarization of rat microglia to M2 phenotype, which was associated with anti-inflammatory and neuroprotective effects (Yuan et al., 2024).

Therefore, modulating histone acetylation through either enhancing or inhibiting HAT activity, as well as targeting HDACs to regulate histone deacetylation, has been shown to provide neuroprotection against cerebral ischemia-induced brain damage. These strategies help restore transcriptional homeostasis and counteract ischemia-associated gene expression dysregulation.

## 4 Targeting epigenetic modifying enzymes as therapeutic strategies for ischemic stroke

Epigenetic drugs target enzymes involved in the regulation of epigenetic changes (Singh et al., 2023) or by inhibiting various enzymes through articulated proteins involved in epigenetic inheritance (Sahafnejad et al., 2023) to maintain normal cellular function. In the past, DNA methylation inhibitors and HDAC inhibitors were considered the most promising drugs (DeWoskin and Million, 2013). Most FDA-approved epigenetic drugs are used in cancer treatment (Davalos and Esteller, 2023). However, they also provide new directions for the treatment of IS.

### 4.1 DNMT inhibitors

No approved central nervous system (CNS) drugs directly affect DNA methylation (Toth, 2021). However, DNA methylation inhibitors have demonstrated efficacy in IS. Decitabine (5-aza-2′-deoxycytidine or AzadC) and azacitidine (5-azacytidine or AzaC) are prototype cytidine analogs, known as DNMT inhibitor (Creusot et al., 1982; Laranjeira et al., 2023). Pharmacological inhibition of DNA methyltransferases by decitabine alters epigenetic levels. A study showed that decitabine-induced cytotoxicity was associated with changes in DNMT1 and DNMT3A expression, which may have affected brain function (Yang J. et al., 2017). The administration of decitabine in the brain ventricles before ischemia was observed to significantly reduce ischemic damage and decrease neurobehavioral deficits in ischemic models (Endres et al., 2000). Furthermore, it improved ischemia-induced neuronal death by inhibiting DNA methylation after MCAO (Hwang et al., 2017) and reduced astrocyte proliferation (Zhang et al., 2022). Decitabine treatment combined with task-specific training significantly downregulated DNMT1, DNMT3a, and DNMT3b protein expression, and promoted motor function recovery in rats (Choi et al., 2018). Additionally, decitabine may have ameliorated cerebral ischemia by inhibiting the complexes formed by DNMT1 with other proteins (Guida et al., 2024). An *in vivo* research showed that 5-Aza-CdR increased hypoxia/ischemia tolerance in mouse neuronal HT22 cells by inducing autophagy (Qi et al., 2019). Azacitidine can also induced neurogenic differentiation in human adipose tissue stromal cells, improving functional recovery in MCAO rats with motor deficits (Kang et al., 2003).

RG108, a non-nucleoside DNMT inhibitor, inhibits the increase in N-methyl-D-aspartate (NMDA)-induced 5mC-positive cell numbers in the brain after IS and protects against NMDA-induced neuronal cell death. Although this study does not investigate the effect of RG108 on the area of cerebral infarction after MCAO/R, it may be associated with the severity, time course, and brain area of the ischemic condition (Asada et al., 2020).

Zebularine is a novel DNMT inhibitor. A study claimed that zebularine maintained BBB integrity in MCAO mice by increasing the expression of zona occludens-1 (ZO-1) and vascular endothelial (VE) calmodulin (Zeng et al., 2022). Knockdown of DNMT1, DNMT3a, and DNMT3b in Neuro2a cells revealed that it is likely that at least two or all DNMTs functionally cooperated in the activation of DNA methylation after glutamatergic excitotoxicity and that zebularine prevented glutamatergic excitotoxicity in Neuro2a cells and primary cortical neurons by inhibiting DNA methylation in neurons after cerebral ischemia-induced neuronal cell death (Asada et al., 2022). In summary, DNMT inhibitors have been shown to mitigate stroke severity, delay ischemic brain damage, and provide neuroprotection against IS.

### 4.2 TET activators

Vitamin C, also known as ascorbic acid, serves as a TET activator, largely due to its cofactor function in Fe(II) and 2-oxoglutarate-dependent dioxygenase reactions (Liu et al., 2022). This enhances the catalytic activity of Tet dioxygenases in the oxidation of 5mC (Yin et al., 2013) and stimulates the expression of TET-associated genes, especially those involved in axonal guidance and ion channel regulation (Zhu et al., 2016). Research has indicated that ascorbic acid treatment following stroke increases TET3 activity and boosts 5hmC levels, which are instrumental in reducing infarct size after focal ischemia (Morris-Blanco et al., 2022).

### 4.3 HAT activators/inhibitors

Lys-CoA, a pioneering HAT inhibitor, has emerged as a selectivity enhancer for the p300 and PCAF HAT enzymes (Lau et al., 2000). Its impact on synaptic plasticity is particularly noteworthy, as it has demonstrated the ability to enhance this critical function in the synaptic architecture of mice (Marek et al., 2011), a feature that is indispensable for learning and memory dynamics. Garcinol, extracted from the rind of *Garcinia morella*, is a polyisoprenylated benzophenone derivative with remarkable inhibitory effects on p300 and PCAF (Balasubramanyam et al., 2004). Its therapeutic potential is further underscored by its capacity to mitigate the inflammatory and oxidative stress responses triggered by ischemia/reperfusion (I/R) injuries, both in cellular models and within living organisms (Kang et al., 2020), positioning it as a promising candidate for the prevention of cerebral ischemic reperfusion injury. In addition, modulating the function of small-molecule regulatory enzymes may contribute to the treatment of IS. Compound A485, a small-molecule CBP/p300 inhibitor (Lasko et al., 2017), exerts neuroprotective effects by inhibiting the formation of Kla proteins. This action is pivotal in alleviating the devastating consequences of IS in mice, including a reduction in neuronal mortality and deactivation of glial cells, which consequently reduces brain damage (Xiong et al., 2024).

TTK21, in its activated state as a small-molecule activator of CBP/p300, achieves a new height of efficacy when complexed with carbon spheres (CSP) to form CSP-TTK21. CSP-TTK21 also functions as an activator of CBP/p300 (Chatterjee et al., 2013). This innovative compound not only easily navigates the BBB, but also does so without incurring significant toxicity, making it a valuable asset in the realm of neuroregeneration and enhancement of long-term memory (Chatterjee et al., 2013).

### 4.4 HDAC inhibitors

HDAC inhibitors are primarily categorized into four classes: hydroxamic acids (Trichostatin A [TSA] and suberoylanilide hydroxamic acid [SAHA]), cyclic ketones (such as trapoxins), short-chain fatty acids (sodium butyrate, phenylbutyrate, and valproic acid), and benzamide derivatives (Kazantsev and Thompson, 2008). Given the success of HDAC inhibitors in cancer treatment, researchers have been exploring the potential of these drugs for IS therapy, and some HDAC inhibitors have shown efficacy in treating IS models. Studies using rat stroke models have demonstrated the neuroprotective effects of HDAC inhibitors in ischemic brain injury (Chuang et al., 2009; Faraco et al., 2006). The pan-HDAC inhibitor SAHA has been reported to enhance neuroplasticity in surviving neurons in the peri-infarct area (Tang Y. et al., 2017). When administered during early reperfusion in hypertensive rats following transient middle cerebral artery occlusion (tMCAO), SAHA significantly reduced cerebral infarct volume, attenuated microglial activation, and protected the BBB, thereby exerting potent cerebrovascular protective effects (Díaz-Pérez et al., 2024). TSA, a hydroxamic acid class of HDAC inhibitor, has been shown to increase histone H3 acetylation after permanent middle cerebral artery occlusion (pMCAO), leading to improvements in motor, sensory, and reflex performance in rats (Kim et al., 2007). Additionally, TSA ameliorates IS by reducing autophagy and lysosomal dysfunction in the ischemic penumbra neurons (Lingling et al., 2022).

Sodium butyrate, a short-chain fatty acid-based HDAC inhibitor, exhibits neuroprotective effects on IS. Intranasal administration of sodium butyrate to rats 1h after MCAO ameliorated MCAO-induced apoptosis (Zhou Z. et al., 2021). Sodium butyrate reduced glial fibrillary acidic protein (GFAP) levels in the serum of MCAO rats and decreased BBB permeability (Park and Sohrabji, 2016). Moreover, sodium butyrate promoted the LPS-induced microglia to change from pro-inflammatory to anti-inflammatory, which attenuated microglia-mediated neuroinflammation and possessed a powerful anti-inflammatory effect (Patnala et al., 2017). In addition, sodium butyrate inhibited systemic inflammatory responses by modulating regulatory T cell levels and related inflammatory pathways, demonstrating neuroprotective effects in a mouse model of diabetic stroke and ameliorating brain injury (Li et al., 2023).

Valproic acid (VPA) is another short-chain fatty acid-based HDAC inhibitor. In a vivo study revealed that treatment with VPA significantly reduced TUNEL-positive cells in the ischemic border zone of the brain tissue of MCAO/R rats and attenuated ischemia-reperfusion injury in the rat brain by inhibiting oxidative stress and inflammation (Suda et al., 2013). Low-dose VPA treatment did not alter cerebral infarct volume but partially promoted the polarization of microglia toward the anti-inflammatory M2 type in the peri-infarct cortex after 3 days of VPA treatment. The number of microglia within the peri-infarct cortex of MCAO/R rat brains was significantly reduced at 7 days. Moreover, a central effect of VPA on microglial morphology was observed on days 2 and 7, which may have been related to HDAC inhibition-mediated suppression of galectin-3 production (Kuo et al., 2021). Cerebral ischemia induces glial scarring. Both *in vitro* and *in vivo* experiments have demonstrated that VPA inhibits HDAC and induces heat-shock protein 70.1B (Hsp70.1B) to produce neuroprotection and inhibit glial scarring during recovery from IS (Gao et al., 2022). An *in vitro* experiment revealed that VPA treatment attenuated apoptosis in OGD-induced BV-2 cells and ameliorated OGD-induced microglial injury (Li et al., 2020). *In vivo*, a transient global cerebral ischemic-reperfusion injury model was constructed with gerbils, and *in vitro* hypoxia-hypoxia treatment for hippocampal neuronal injury showed that VPA attenuated ischemia-reperfusion injury-induced cellular pyroptosis and hippocampal neuronal injury (Zhu et al., 2019). VPA exerts neuroprotective effects, which may be due to its ability to penetrate the BBB (Wang et al., 2011).

Additionally, specific HDAC inhibitors, such as the novel HDAC6 inhibitor tubastatin A (TubA), improved functional recovery, reduced brain infarct volume, and ameliorated neuronal cell death in MCAO rats (Wang et al., 2016a). HDAC6 serves as a deacetylase for macrophage migration inhibitory factor (MIF), and its inhibition by TubA treatment markedly enhances MIF acetylation in methylnitronitrosoguanidine-induced neuro2a cells. This increase in MIF acetylation plays a critical role in mediating the neuroprotective effects of HDAC6 inhibitors in IS (Hu et al., 2022). MI-192, an inhibitor of HDAC2 and HDAC3, reduced cerebral infarct core volume and apoptosis, partially restored functional symmetry in forelimb use, and exerted a neuroprotective effect on the mouse brain in a PTS mouse model (Demyanenko et al., 2020c).

Sirtinol, a broad-spectrum SIRT inhibitor, acts as an antagonist of SIRT1, induces microglial activation under OGD/R conditions (Liao et al., 2023) and exacerbates ischemic injury (Hernández-Jiménez et al., 2013). SIRT1 overexpression enhances the deacetylation of SIRT3, boosting its activity and improving neurological damage caused by cerebral ischemia-reperfusion injury and mitochondrial dysfunction. However, the neuroprotective effects of SIRT1 are partially offset by the SIRT3 inhibitor 3-(1H-1,2,3-triazole-4-yl) pyridine(3-TYP) (Chen M. et al., 2024). The “SIRT1/SIRT3 activity” axis offers potential therapeutic prospects for IS-related diseases. Nonetheless, high doses of Sirt3 inhibitors exacerbate ischemic injury, and the off-target effects of Sirt1 suggest that targeting Sirt3 may not be an ideal approach. Instead, strategies aimed at promoting increased SIRT1 expression may serve as neuroprotective strategies (Verma et al., 2019). In an *in vivo* ischemia-reperfusion injury model, treatment with the SIRT2 inhibitors AK1 and AGK2 has been shown to reduce infarct size in the ipsilateral hemisphere, improve neurological outcomes, and decrease apoptosis-induced cell death in in vitro OGD models (She et al., 2018). AGK2 (Jiao et al., 2020) and another SIRT2 inhibitor, AK7, play a positive role in the inhibition of neuroinflammation (Wu et al., 2020). Additionally, AK7 has been found to regulate microglial polarization (Wu et al., 2020). After cerebral ischemic injury, with specific P38 activation, the administration of AK7 demonstrated concentration-dependent effects, improving outcomes in cerebral ischemia (Wu et al., 2018). Recent research has highlighted the SIRT5 inhibitor MC3482, which upregulates succinylation levels of annexin-A1, promoting its membrane recruitment and extracellular secretion. This mechanism alleviates neuroinflammation induced by microglia after IS and improves the long-term neurological function in mice with stroke (Xia et al., 2024). Furthermore, in rat CI/RI and H19-7 hippocampal neuronal injury models, lentiviral transfection of SIRT5 not only improved the range of ischemia-induced neurological injuries, but also prevented cell ferroptosis (Li J. et al., 2024).

## 5 Natural compounds as epigenetic enzyme regulators in IS

Epigenetic enzymes, which are key regulators of IS pathology, have emerged as promising therapeutic targets. Natural compounds with the ability to modulate these enzymes have attracted considerable attention, offering novel strategies for therapeutic (Table 1).

**TABLE 1:** Natural compounds targeting epigenetic enzymes in IS.

Phytocompounds	Source	*In vivo* model	*In vitro* model	Targets	References
Curcumin	*Turmeric rhizomes*	MCAO/R^a^-induced C57BL/6N mice	OGD^b^-induced primary neurons	p300/CBP	Yildirim et al. (2014)
		MCAO/R-induced SD rats	—	SIRT1	Miao et al. (2016)
Tetrahydrocurcumin	*Curcumin*	MCAO/R-induced C57BL/6J mice	—	DNMTs	Mondal et al. (2019)
Resveratrol	*Grapes, red wine, blueberries and peanuts, red wine and other foods*	IPC^c^- induced SD rats	OGD/R^d^-induced microglia, primary cortical neurons	SIRT1	Liao et al. (2023), Park et al. (2012), Wan et al. (2016)
		—	LPS^e^-induced ARPE-19 cells	DNMT, SIRT1	Maugeri et al. (2018)
		MCAO/R-induced C57BL/6 mice	OGD-induced primary cortical neurons	HDAC	Lanzillotta et al. (2013)
EGCG	*Green tea*	—	OGD-induced primary cortical neurons	HAT	Faggi et al. (2019)
Berberine	*Buttercup, berberidaceae, and rutaceae families*	MCAO/R-induced C57BL/6J mice	OGD/R-induced primary neurons and astrocytes co-culture	METTL3	Hu et al. (2024)
Wogonin	*Scutellaria baicalensis*	MCAO/R-induced SD rats	OGD/R-induced HT-22 cells	SIRT1	Cheng et al. (2024)
Apigenin	*Asteraceae plants*	MCAO/R-induced SD rats	—	HDAC	Tu et al. (2017)
Quercetin	*Fruits, vegetables, and nuts*	MCAO/R-induced SD rats	—	SIRT1	Yang et al. (2022)
Icariside II	*Epimedium brevicornum Maxim*	PSD^f^- induced C57BL/6J mice	—	SIRT6	Gao et al. (2025)
Trilobatin	*Lithocarpus polystachyus*	MCAO/R-induced SD rats	OGD/R-induced astrocytes	SIRT3	Gao et al. (2020)
		MCAO/R-induced SD rats	—	SIRT6/7	Huang et al. (2022)
Astragaloside IV	*Astragalus membranaceus*	MCAO/R-induced SD rats	—	SIRT1	Shi et al. (2021)
		MCAO/R-induced SD rats	OGD/R-induced HUVECs cells	SIRT7	Ou et al. (2023)
Cycloastragenol	*Astragalus Radix*	MCAO/R-induced C57BL/6 mice	—	SIRT1	Li and Sui (2020)
Forsythoside B	*Forsythiae Fructus*	MCAO/R-induced SD rats	—	SIRT1	Li Q. et al. (2024)
Pterostilbene	*Sandalwood*	MCAO/R-induced C57BL/6N mice	OGD/R--induced primary microglia	HDAC3	Chen Y. et al. (2024)

^a^ MCAO/R, Middle cerebral artery occlusion/reperfusion.

^b^ OGD, Oxygen-glucose deprivation.

^c^ IPC, Ischemic preconditioning.

^d^ OGD/R, Oxygen-glucose deprivation/reperfusion.

^e^ LPS, Lipopolysaccharide.

^f^ PSD, Poststroke depression.

### 5.1 Curcumin

Curcumin, a polyphenol derived from *turmeric rhizomes*, mitigates neuroinflammation and oxidative stress associated with IS through various mechanisms, including epigenetic modifications (Hatami et al., 2019). A molecular docking study has demonstrated that curcumin interacts with DNMT1 and regulates genomic DNA methylation by inhibiting its activity (Liu et al., 2009). At specific concentrations, curcumin reduces global DNA methylation to levels comparable to those of decitabine, underscoring its potential as an effective DNA hypomethylating agent (Fu and Kruzrock, 2010). Additionally, curcumin-related compounds, such as demethoxycurcumin and bisdemethoxycurcumin, influence epigenetic characteristics (Liu et al., 2011a). These compounds suppress DNMT1 activity, induce demethylation in the Wnt inhibitor-1 (WIF-1) promoter region (Liu et al., 2011b), and exert neuroprotective effects by modulating the canonical Wnt pathway (Ashrafizadeh et al., 2020).

Curcumin is a specific inhibitor of HAT p300/CBP (Dekker and Haisma, 2009). In a mouse model of 5-min ischemic preconditioning, curcumin reduced the acetylation levels of histones H3 and H4 in a dose-dependent manner, thereby diminishing neuronal ischemic tolerance induced by ischemic preconditioning (Yildirim et al., 2014). One of the primary metabolites of curcumin, tetrahydrocurcumin (THC), downregulates the expression of DNMT1 and DNMT3A proteins and genes in the mitochondria of CI/RI mice. It also reverses the sharp increase in the overall brain DNMT activity, demonstrating the potential of epigenetic targeted therapy (Mondal et al., 2019). Computational studies comparing curcumin, curcuminoid compounds (demethoxycurcumin and bisdemethoxycurcumin), and FDA-approved HDAC inhibitors for drug similarity and toxicity revealed that curcumin and its derivatives interact with the active sites of HDACs, exhibiting inhibitory effects similar to those of standard HDAC inhibitors (Anandaradje et al., 2024). Moreover, these natural compounds exhibit no reported toxicity or mutagenicity and possess a higher lethal dose (LD50) than chemical drugs (Anandaradje et al., 2024). A molecular docking study of curcumin with the human HDAC8 enzyme further predicted its inhibitory activity, revealing a stable 3D inhibitor-enzyme complex. Notably, curcumin derivatives exhibited superior inhibitory efficiency compared to the HDAC inhibitor sodium butyrate (Bora-Tatar et al., 2009). Furthermore, curcumin mitigates IS-induced brain injury by upregulating SIRT1 expression (Miao et al., 2016). Collectively, these findings suggest that curcumin may serve as a dual inhibitor of HAT and HDAC, exerting epigenetic regulatory effects that modulate IS pathogenesis.

### 5.2 Resveratrol

Resveratrol, a naturally occurring polyphenol, is present in foods, such as grapes, red wine, blueberries, peanuts, and other delicacies (Abdelsalam et al., 2023). It is widely recognized as a potent SIRT1 activator (Shin et al., 2012). Activation of SIRT1 by resveratrol triggers deacetylation of downstream targets, promoting IS cell survival and inhibiting apoptosis (Owjfard et al., 2024). A brief period of ischemia prior to a prolonged ischemic event has been observed to mitigate the adverse effects on the brain (Dirnagl et al., 2003). This endogenous protective mechanism is known as ischemic preconditioning (IPC). The long-term window of ischemic tolerance induced by IPC can last up to 1 week (Khoury et al., 2016). Notably, resveratrol preconditioning (RPC) extends this window of cerebral ischemic tolerance to 2 weeks in mice (Khoury et al., 2019). A study has shown that RPC mimics IPC in the hippocampal CA1 region by significantly increasing SIRT1 activity, and blocking SIRT1 activation during reperfusion after RPC negates the neuroprotective effects of RPC (Raval et al., 2006). Similarly, David Della-Morte et al. confirmed that RPC targets mitochondrial uncoupling protein 2 (UCP2) via the SIRT1 pathway, altering mitochondrial function and providing IPC-like protection against cerebral ischemic injury in rats (Della-Morte et al., 2009).Additionally, resveratrol upregulates SIRT1 expression, promotes neurite growth and synaptogenesis, and reduces neuronal damage following OGD/R (Tang F. et al., 2017). A key mechanism by which resveratrol attenuates inflammation is through SIRT1 activation. A recent study suggests that resveratrol-mediated SIRT1 activation may suppress OGD/R-induced microglial activation and inflammatory responses via the Shh/Gli-1 signaling pathway (Liao et al., 2023). Moreover, resveratrol is thought to indirectly enhance SIRT1 activity through cAMP and AMPK signaling (Park et al., 2012), modulating the cAMP/AMPK/SIRT1 pathway to reduce energy expenditure during ischemia and provide neuroprotection (Wan et al., 2016).

Beyond its role as a SIRT1 activator, resveratrol also functions as a DNMT inhibitor by suppressing DNMTs activity and expression (Ionescu et al., 2021). However, its effects on DNMT regulation appear to be context-dependent. In LPS-induced human retinal pigment epithelial cells, resveratrol reduces DNMT1 expression and total DNMT activity. Interestingly, under inflammatory conditions, it restores DNMT1 expression and total DNMT activity, possibly via SIRT1-mediated DNMT regulation (Maugeri et al., 2018). Chemical modifications of resveratrol have yielded more potent and selective inhibitors of HDAC1 and HDAC2 (Urias et al., 2022). In both *in vivo* and *in vitro* model of cerebral ischemia, combining HDAC inhibitors with very low doses of resveratrol exerted synergistic neuroprotective effects, significantly prolonging neuroprotection (Lanzillotta et al., 2013).

### 5.3 Tea polyphenols

Tea polyphenols are a collection of bioactive compounds including catechins, epicatechins, and the notably potent epigallocatechin-3-gallate (EGCG). These catechin polyphenols influence DNA methylation through both direct and indirect inhibition of DNMTs (Yang et al., 2008). EGCG, a primary constituent of *green tea*, has been demonstrated to impede DNMT-driven methylation processes *in vitro*. This intervention results in demethylation of promoter CpG islands, subsequently reactivating genes that have been silenced by methylation (Fang et al., 2007). As a DNMT inhibitor, EGCG reduces 5mC, DNMT1, DNMT3A, and DNMT3B protein levels in a dose-dependent manner, effectively reshaping the DNA methylation profile (Nandakumar et al., 2011). Research indicates that EGCG protects neurons from ischemic injury by modulating autophagy in a phosphorylation-dependent manner in both *in vivo* and *in vitro* models of IS (Wang et al., 2022). Pretreatment of cortical neurons exposed to OGD with a polyphenol-rich micronutrient mixture significantly reduced the acetylation of histones H3 and H4, mimicking the effects of a HAT inhibitor (Faggi et al., 2019). Furthermore, EGCG regulates the balance between HATs and HDACs in inflammatory signaling pathways by reducing the binding affinity of p300/CBP while enhancing the recruitment of HDAC3 (Choi et al., 2009).

### 5.4 Berberine

Berberine (BBR), a compound commonly found in plants of the *Buttercup, Berberidaceae, and Rutaceae* families, exhibits properties similar to DNMT inhibitors by significantly downregulating DNMT1 and DNMT3B levels (Wang X. et al., 2024). BBR also influences growth arrest and apoptosis in cells where HDAC activity is inhibited (Kalaiarasi et al., 2016). In U266 cells treated with BBR, notable alterations in epigenetic regulators were observed, with at least a 1.5-fold increase. These include upregulation of histone acetyltransferases (CREBBP, EP300, and HAT1) and histone deacetylases (SIRT3 and HDAC5/9), along with downregulation of HDAC2/8 and DNMT1/3B (Wang et al., 2016b). After stroke, the brain transcriptome undergoes significant remodeling, and berberine has been shown to exert neuroprotective effects against ischemic brain injury. This neuroprotection is mediated by the m6A methyltransferase METTL3 in mouse astrocytes (Hu et al., 2024).

### 5.5 Flavonoids

Wogonin, a flavonoid naturally extracted from the roots of *Scutellaria baicalensis*, effectively activates the AMPK/SIRT1 signaling pathway, leading to the upregulation of both SIRT1 expression and activity. Once activated, SIRT1 exerts neuroprotective effects by deacetylating and downregulating NLRP3 inflammasome-associated molecules, thereby mitigating inflammation and alleviating cerebral ischemia-reperfusion injury (Cheng et al., 2024).

Apigenin. a widely distributed flavonoid formally classified as a flavone, is primary found in plants of the *Asteraceae* family. A study has shown that apigenin significantly reduces hippocampal HDAC levels in rats subjected to MCAO, restores acetylated histones H3 and H4 levels, and ameliorates memory deficits (Tu et al., 2017).

Quercetin, a flavonoid and naturally occurring polyphenol, is most abundantly found in onions. It has been identified as a potential small-molecule modulator of SIRT for IS treatment (Bai et al., 2018). Quercetin exerts reparative effects on brain damage by inhibiting the SIRT1 signaling pathway, thereby improving BBB permeability and reducing reactive ROS generation in rats with cerebral ischemia-reperfusion injury (Yang et al., 2022).

Icariside II is a natural flavonoid glycoside extracted from *Epimedium brevicornum Maxim*. As a naturally occurring neuroprotective agent, it exhibits potent Sirt6-inducing activity. A recent study demonstrated that icariside II functions as a SIRT6 activator by directly binding to and enhancing SIRT6 activity. Moreover, it alleviates post-stroke depression in mice by regulating the microbiota-gut-brain axis (Gao et al., 2025).

Trilobatin is a flavonoid extracted from *Lithocarpus polystachyus*. As a novel natural SIRT3 activator, trilobatin not only directly binds to SIRT3 but also increases its expression and activity, thereby inhibiting brain I/R-induced neuroinflammation and oxidative damage (Gao et al., 2020). In MCAO/R-induced rats, trilobatin significantly upregulated the protein expression of SIRT6 and SIRT7, while having no effect on SIRT1–SIRT5 expression. Moreover, trilobatin directly binds to SIRT7 and enhances its expression on day 28 post-injury. By modulating the SIRT7/VEGFA signaling pathway, trilobatin promotes angiogenesis following cerebral ischemic injury in rats (Huang et al., 2022).

### 5.6 Other natural compounds

Astragaloside IV, a major constituent of *Astragalus membranaceus*, enhances SIRT1 activity by upregulating its expression and exerts neuroprotective effects through modulating the SIRT1/Mapt pathway in a rat model of CI/RI (Shi et al., 2021). Cycloastragenol, the active metabolite of astragaloside IV isolated from *Astragalus Radix*, upregulates SIRT1 expression and mitigates apoptosis and neuroinflammation caused by IS (Li and Sui, 2020). Furthermore, astragaloside IV not only binds to SIRT7 but also increases the expression of SIRT7, which promotes angiogenesis via the SIRT7/VEGFA signaling pathway to facilitate post-stroke brain tissue repair (Ou et al., 2023).

Forsythoside B, a bioactive compound derived from *Forsythiae Fructus*, has been shown to attenuate oxidative stress and upregulate SIRT1 expression in MCAO/R rats. Notably, the protective effects of Forsythoside B against inflammation and oxidative stress were abolished upon SIRT1 knockdown. These findings suggest that Forsythoside B exerts neuroprotective and anti-inflammatory effects in CI/RI through modulation of SIRT1 signaling (Li Q. et al., 2024).

Pterostilbene, a resveratrol derivative originally extracted from *sandalwood*, has been shown to inhibit the expression and activity of HDAC3 in ischemic brain tissue in an MCAO/R-induced mouse model. Moreover, pterostilbene mitigates OGD/R-induced microglial injury and suppresses the production of pro-inflammatory molecules by upregulating HDAC3/Nrf1 signaling in microglia, thereby ameliorating neurological dysfunction and neuroinflammation following IS (Chen Y. et al., 2024).

## 6 Conclusion and future perspectives

Using two groups of epigenetic-modifying enzymes -DNMT and TET, HAT and HDAC-as examples, this paper reviews their roles in IS and the therapeutic potential of targeting these enzymes. As we have explored the role of epigenetic-modifying enzymes in regulating epigenetic mechanisms, new targets for treating IS have emerged. Not only do DNMT inhibitors, TET activators, HDAC inhibitors, and HAT activators/inhibitors show neuroprotective effects, but natural compounds have also shown promising results. However, the current studies are mainly based on *in vitro* experiments and animal models, and the results of these studies, although encouraging, do not directly translate into clinical efficacy. Moreover, we cannot conclude whether these drugs can function safely and effectively in patients with clinical stroke, and many challenges remain.

## Glossary

5caC: 5-carboxylcytosine
5fC: 5-formylcytosine
5hmC: 5-hydroxymethylcytosine
5mC: 5-methylcytosine
AMPK: AMP-activated protein kinase
BBB: Blood-brain barrier
CBP: CREB-binding protein
CREB: cAMP-response element binding protein
CI/RI: Cerebral ischemia/reperfusion injury
CPG: Cytosine-phosphate-Guanine
CSP: Carbon spheres
DNMT: DNA methyltransferase
ECGC: Epigallocatechin-3-gallate
HAT: Histone acetyltransferase
Hcy: Homocysteine
HDAC: Histone deacetylase
IPC: Ischemic preconditioning
IS: Ischemic Stroke
LPS: Lipopolysaccharide
MCAO: Middle cerebral artery occlusion
MCAO/R: Middle cerebral artery occlusion/reperfusion
NCX1: Na+/Ca2+exchanger-1
OGD: Oxygen-glucose deprivation
OGD/R: Oxygen-glucose deprivation/reperfusion
PCAF: P300/CBP-associated factor
PTM: Post-translational modification
PTS: Photothrombotic stroke
RPC: Resveratrol preconditioning
SIRT: Sirtuin
TET: Ten-eleven translocation
VPA: Valproic acid

## References

[B1] Abdelsalam S. A. Renu K. Zahra H. A. Abdallah B. M. Ali E. M. Veeraraghavan V. P. (2023). Polyphenols mediate neuroprotection in cerebral ischemic stroke-an update. Nutrients 15, 1107. 10.3390/nu15051107 36904106 PMC10005012

[B2] Aldridge A. I. West A. E. (2024). Epigenetics and the timing of neuronal differentiation. Curr. Opin. Neurobiol. 89, 89102915. 10.1016/j.conb.2024.102915 PMC11611672 39277975

[B3] Anandaradje A. Kalita B. Coumar M. S. Selvarajan S. (2024). “Molecular docking of curcumin and curcuminoids as human Zn(+) dependent histone deacetylase (HDAC) enzyme inhibitors,” in In silico pharmacol, 1247. 10.1007/s40203-024-00221-4 PMC11133269 38817777

[B4] Antunes C. Da Silva J. D. Guerra-Gomes S. Alves N. D. Ferreira F. Loureiro-Campos E. (2021). Tet3 ablation in adult brain neurons increases anxiety-like behavior and regulates cognitive function in mice. Mol. Psychiatry 26, 261445–261457. 10.1038/s41380-020-0695-7 32103150

[B5] Armour E. M. Thomas C. M. Greco G. Bhatnagar A. Elefant F. (2023). Experience-dependent Tip60 nucleocytoplasmic transport is regulated by its NLS/NES sequences for neuroplasticity gene control. Mol. Cell Neurosci. 127, 127103888. 10.1016/j.mcn.2023.103888 PMC11337217 37598897

[B6] Asada M. Hayashi H. Murakami K. Kikuiri K. Kaneko R. Yuan B. (2020). Investigating the relationship between neuronal cell death and early DNA methylation after ischemic injury. Front. Neurosci. 14, 14581915. 10.3389/fnins.2020.581915 PMC7591788 33177984

[B7] Asada M. Hayashi H. Takagi N. (2022). Possible involvement of DNA methylation and protective effect of zebularine on neuronal cell death after glutamate excitotoxity. Biol. Pharm. Bull. 45, 45770–45779. 10.1248/bpb.b22-00147 35650104

[B8] Ashrafizadeh M. Ahmadi Z. Mohamamdinejad R. Yaribeygi H. Serban M. C. Orafai H. M. (2020). Curcumin therapeutic modulation of the Wnt signaling pathway. Curr. Pharm. Biotechnol. 21, 211006–211015. 10.2174/1389201021666200305115101 32133961

[B9] Atsumi Y. Iwata R. Kimura H. Vanderhaeghen P. Yamamoto N. Sugo N. (2024). Repetitive CREB-DNA interactions at gene loci predetermined by CBP induce activity-dependent gene expression in human cortical neurons. Cell Rep. 43, 43113576. 10.1016/j.celrep.2023.113576 38128530

[B10] Bai X. Yao L. Ma X. Xu X. (2018). Small molecules as SIRT modulators. Mini Rev. Med. Chem. 18, 181151–181157. 10.2174/1389557516666160620095103 27334466

[B11] Balasubramanyam K. Altaf M. Varier R. A. Swaminathan V. Ravindran A. Sadhale P. P. (2004). Polyisoprenylated benzophenone, garcinol, a natural histone acetyltransferase inhibitor, represses chromatin transcription and alters global gene expression. J. Biol. Chem. 279, 27933716–27933726. 10.1074/jbc.M402839200 15155757

[B12] Baltan S. Bachleda A. Morrison R. S. Murphy S. P. (2011). Expression of histone deacetylases in cellular compartments of the mouse brain and the effects of ischemia. Transl. Stroke Res. 2, 2411–2423. 10.1007/s12975-011-0087-z PMC3182145 21966324

[B13] Bardai F. H. Price V. Zaayman M. Wang L. D'Mello S. R. (2012). Histone deacetylase-1 (HDAC1) is a molecular switch between neuronal survival and death. J. Biol. Chem. 287, 28735444–28735453. 10.1074/jbc.M112.394544 PMC3471765 22918830

[B14] Bayer C. Pitschelatow G. Hannemann N. Linde J. Reichard J. Pensold D. (2020). DNA methyltransferase 1 (DNMT1) acts on neurodegeneration by modulating proteostasis-relevant intracellular processes. Int. J. Mol. Sci. 21, 5420. 10.3390/ijms21155420 32751461 PMC7432412

[B15] Beaver M. Bhatnagar A. Panikker P. Zhang H. Snook R. Parmar V. (2020). Disruption of Tip60 HAT mediated neural histone acetylation homeostasis is an early common event in neurodegenerative diseases. Sci. Rep. 10, 1018265. 10.1038/s41598-020-75035-3 PMC7588445 33106538

[B16] Berkyurek A. C. Suetake I. Arita K. Takeshita K. Nakagawa A. Shirakawa M. (2014). The DNA methyltransferase Dnmt1 directly interacts with the SET and RING finger-associated (SRA) domain of the multifunctional protein Uhrf1 to facilitate accession of the catalytic center to hemi-methylated DNA. J. Biol. Chem. 289, 289379–289386. 10.1074/jbc.M113.523209 PMC3879560 24253042

[B17] Bernstein C. (2022). DNA methylation and establishing memory. Epigenet Insights 15, 1525168657211072499. 10.1177/25168657211072499 PMC8793415 35098021

[B18] Bertogliat M. J. Morris-Blanco K. C. Vemuganti R. (2020). Epigenetic mechanisms of neurodegenerative diseases and acute brain injury. Neurochem. Int. 133, 133104642. 10.1016/j.neuint.2019.104642 PMC8074401 31838024

[B19] Bhaumik S. R. Smith E. Shilatifard A. (2007). Covalent modifications of histones during development and disease pathogenesis. Nat. Struct. Mol. Biol. 14, 141008–141016. 10.1038/nsmb1337 17984963

[B20] Bora-Tatar G. Dayangaç-Erden D. Demir A. S. Dalkara S. Yelekçi K. Erdem-Yurter H. (2009). Molecular modifications on carboxylic acid derivatives as potent histone deacetylase inhibitors: activity and docking studies. Bioorg Med. Chem., 175219–175228. 10.1016/j.bmc.2009.05.042 19520580

[B21] Bourc'his D. Xu G. L. Lin C. S. Bollman B. Bestor T. H. (2001). Dnmt3L and the establishment of maternal genomic imprints. Science, 2942536–2942539. 10.1126/science.1065848 11719692

[B22] Brookes R. L. Crichton S. Wolfe C. D. A. Yi Q. Li L. Hankey G. J. (2018). Sodium valproate, a histone deacetylase inhibitor, is associated with reduced stroke risk after previous ischemic stroke or transient ischemic attack. Stroke 49, 4954–4961. 10.1161/strokeaha.117.016674 PMC5753817 29247141

[B23] Carrizzo A. Iside C. Nebbioso A. Carafa V. Damato A. Sciarretta S. (2022). SIRT1 pharmacological activation rescues vascular dysfunction and prevents thrombosis in MTHFR deficiency. Cell Mol. Life Sci. 79410, 410. 10.1007/s00018-022-04429-5 PMC9276577 35821533

[B24] Cavalieri V. (2021). The expanding constellation of histone post-translational modifications in the epigenetic landscape. Genes (Basel) 12, 1596. 10.3390/genes12101596 34680990 PMC8535662

[B25] Chatterjee S. Mizar P. Cassel R. Neidl R. Selvi B. R. Mohankrishna D. V. (2013). A novel activator of CBP/p300 acetyltransferases promotes neurogenesis and extends memory duration in adult mice. J. Neurosci. 33, 3310698–3310712. 10.1523/jneurosci.5772-12.2013 PMC6618502 23804093

[B26] Chen H. Liu J. Chen M. Wei Z. Yuan J. Wu W. (2024). SIRT3 facilitates mitochondrial structural repair and functional recovery in rats after ischemic stroke by promoting OPA1 expression and activity. Clin. Nutr. 43, 431816–431831. 10.1016/j.clnu.2024.06.001 38870662

[B27] Chen M. Liu J. Wu W. Guo T. Yuan J. Wu Z. (2024). SIRT1 restores mitochondrial structure and function in rats by activating SIRT3 after cerebral ischemia/reperfusion injury. Cell Biol. Toxicol. 4031, 31. 10.1007/s10565-024-09869-2 PMC11106166 38767771

[B28] Chen R. Qian L. Zhang Q. Qin J. Chen X. Xu X. (2025). SMP30 alleviates cerebral ischemia/reperfusion-induced neuronal injury by inhibiting HDAC4/PSD-95 to preserve mitochondrial function. J. Neuropathol. Exp. Neurol. 84, 8459–8473. 10.1093/jnen/nlae095 39254519

[B29] Chen Y. He W. Qiu J. Luo Y. Jiang C. Zhao F. (2024). Pterostilbene improves neurological dysfunction and neuroinflammation after ischaemic stroke via HDAC3/Nrf1-mediated microglial activation. Cell Mol. Biol. Lett. 29114, 114. 10.1186/s11658-024-00634-1 PMC11360871 39198723

[B30] Chen Y. T. Zang X. F. Pan J. Zhu X. L. Chen F. Chen Z. B. (2012). Expression patterns of histone deacetylases in experimental stroke and potential targets for neuroprotection. Clin. Exp. Pharmacol. Physiol. 39, 39751–39758. 10.1111/j.1440-1681.2012.05729.x 22651689

[B31] Cheng J. Fan Y. Q. Jiang H. X. Chen S. F. Chen J. Liao X. Y. (2021). Transcranial direct-current stimulation protects against cerebral ischemia-reperfusion injury through regulating Cezanne-dependent signaling. Exp. Neurol. 345, 345113818. 10.1016/j.expneurol.2021.113818 34324860

[B32] Cheng Z. Tu J. Wang K. Li F. He Y. Wu W. (2024). Wogonin alleviates NLRP3 inflammasome activation after cerebral ischemia-reperfusion injury by regulating AMPK/SIRT1. Brain Res. Bull. 207, 207110886. 10.1016/j.brainresbull.2024.110886 38253131

[B33] Chestnut B. A. Chang Q. Price A. Lesuisse C. Wong M. Martin L. J. (2011). Epigenetic regulation of motor neuron cell death through DNA methylation. J. Neurosci. 31, 3116619–3116636. 10.1523/jneurosci.1639-11.2011 PMC3238138 22090490

[B34] Chialastri A. Sarkar S. Schauer E. E. Lamba S. Dey S. S. (2024). Combinatorial quantification of 5mC and 5hmC at individual CpG dyads and the transcriptome in single cells reveals modulators of DNA methylation maintenance fidelity. Nat. Struct. Mol. Biol. 31, 311296–311308. 10.1038/s41594-024-01291-w PMC12551665 38671229

[B35] Choi D. H. Choi I. A. Lee J. (2022). The role of DNA methylation in stroke recovery. Int. J. Mol. Sci. 23, 10373. 10.3390/ijms231810373 36142283 PMC9499691

[B36] Choi I. A. Lee C. S. Kim H. Y. Choi D. H. Lee J. (2018). Effect of inhibition of DNA methylation combined with task-specific training on chronic stroke recovery. Int. J. Mol. Sci. 19, 2019. 10.3390/ijms19072019 29997355 PMC6073594

[B37] Choi K. C. Jung M. G. Lee Y. H. Yoon J. C. Kwon S. H. Kang H. B. (2009). Epigallocatechin-3-gallate, a histone acetyltransferase inhibitor, inhibits EBV-induced B lymphocyte transformation via suppression of RelA acetylation. Cancer Res. 69, 69583–69592. 10.1158/0008-5472.Can-08-2442 19147572

[B38] Chuang D. M. Leng Y. Marinova Z. Kim H. J. Chiu C. T. (2009). Multiple roles of HDAC inhibition in neurodegenerative conditions. Trends Neurosci. 32, 32591–32601. 10.1016/j.tins.2009.06.002 PMC2771446 19775759

[B39] Creusot F. Acs G. Christman J. K. (1982). Inhibition of DNA methyltransferase and induction of Friend erythroleukemia cell differentiation by 5-azacytidine and 5-aza-2'-deoxycytidine. J. Biol. Chem. 257, 2572041–2572048. 10.1016/s0021-9258(19)68144-5 6173384

[B40] Cui J. Liu N. Chang Z. Gao Y. Bao M. Xie Y. (2020). Exosomal MicroRNA-126 from RIPC serum is involved in hypoxia tolerance in SH-SY5Y cells by downregulating DNMT3B. Mol. Ther. Nucleic Acids 20, 20649–20660. 10.1016/j.omtn.2020.04.008 PMC7210387 32380415

[B41] Cui M. Huang Y. Tian C. Zhao Y. Zheng J. (2011). FOXO3a inhibits TNF-α- and IL-1β-induced astrocyte proliferation:Implication for reactive astrogliosis. Glia 59, 59641–59654. 10.1002/glia.21134 PMC3747776 21294163

[B42] Dai Y. Wei T. Shen Z. Bei Y. Lin H. Dai H. (2021). Classical HDACs in the regulation of neuroinflammation. Neurochem. Int. 150, 150105182. 10.1016/j.neuint.2021.105182 34509559

[B43] Davalos V. Esteller M. (2023). Cancer epigenetics in clinical practice. CA Cancer J. Clin. 73, 73376–73424. 10.3322/caac.21765 36512337

[B44] Dekker F. J. Haisma H. J. (2009). Histone acetyl transferases as emerging drug targets. Drug Discov. Today 14, 14942–14948. 10.1016/j.drudis.2009.06.008 19577000

[B45] Della-Morte D. Dave K. R. DeFazio R. A. Bao Y. C. Raval A. P. Perez-Pinzon M. A. (2009). Resveratrol pretreatment protects rat brain from cerebral ischemic damage via a sirtuin 1-uncoupling protein 2 pathway. Neuroscience 159, 159993–161002. 10.1016/j.neuroscience.2009.01.017 PMC2668125 19356683

[B46] Demyanenko S. Gantsgorn E. Rodkin S. Sharifulina S. (2020). Localization and expression of sirtuins 1, 2, 6 and plasticity-related proteins in the recovery period after a photothrombotic stroke in mice. J. Stroke Cerebrovasc. Dis. 29, 29105152. 10.1016/j.jstrokecerebrovasdis.2020.105152 32912518

[B47] Demyanenko S. Neginskaya M. Berezhnaya E. (2018). Expression of class I histone deacetylases in ipsilateral and contralateral hemispheres after the focal photothrombotic infarction in the mouse brain. Transl. Stroke Res. 9, 9471–9483. 10.1007/s12975-017-0595-6 29218547

[B48] Demyanenko S. Uzdensky A. (2019). Epigenetic alterations induced by photothrombotic stroke in the rat cerebral cortex: deacetylation of histone h3, upregulation of histone deacetylases and histone acetyltransferases. Int. J. Mol. Sci. 20, 2882. 10.3390/ijms20122882 31200484 PMC6627403

[B49] Demyanenko S. V. Dzreyan V. A. Uzdensky A. B. (2020a). Overexpression of HDAC6, but not HDAC3 and HDAC4 in the penumbra after photothrombotic stroke in the rat cerebral cortex and the neuroprotective effects of α-phenyl tropolone, HPOB, and sodium valproate. Brain Res. Bull. 162, 162151–162165. 10.1016/j.brainresbull.2020.06.010 32592806

[B50] Demyanenko S. V. Dzreyan V. A. Uzdensky A. B. (2020b). The expression and localization of histone acetyltransferases HAT1 and PCAF in neurons and astrocytes of the photothrombotic stroke-induced penumbra in the rat brain cortex. Mol. Neurobiol. 57, 573219–573227. 10.1007/s12035-020-01959-6 32506381

[B51] Demyanenko S. V. Nikul V. V. Uzdensky A. B. (2020c). The neuroprotective effect of the HDAC2/3 inhibitor MI192 on the penumbra after photothrombotic stroke in the mouse brain. Mol. Neurobiol. 57, 57239–57248. 10.1007/s12035-019-01773-9 31512115

[B52] Deng Y. Chen D. Gao F. Lv H. Zhang G. Sun X. (2020). Silencing of long non-coding RNA GAS5 suppresses neuron cell apoptosis and nerve injury in ischemic stroke through inhibiting DNMT3B-dependent MAP4K4 methylation. Transl. Stroke Res. 11, 11950–11966. 10.1007/s12975-019-00770-3 31997156

[B53] DeWoskin V. A. Million R. P. (2013). The epigenetics pipeline. Nat. Rev. Drug Discov. 12, 12661–12662. 10.1038/nrd4091 23989788

[B54] Díaz-Pérez A. Pérez B. Manich G. García-Aranda J. Navarro X. Penas C. (2024). Histone deacetylase inhibition by suberoylanilide hydroxamic acid during reperfusion promotes multifaceted brain and vascular protection in spontaneously hypertensive rats with transient ischaemic stroke. Biomed. Pharmacother., 172116287. 10.1016/j.biopha.2024.116287 38382328

[B55] Dirnagl U. Simon R. P. Hallenbeck J. M. (2003). Ischemic tolerance and endogenous neuroprotection. Trends Neurosci. 26, 26248–26254. 10.1016/s0166-2236(03)00071-7 12744841

[B56] Durham B. S. Grigg R. Wood I. C. (2017). Inhibition of histone deacetylase 1 or 2 reduces induced cytokine expression in microglia through a protein synthesis independent mechanism. J. Neurochem. 143214-224, 214–224. 10.1111/jnc.14144 28796285

[B57] Egger G. Jeong S. Escobar S. G. Cortez C. C. Li T. W. Saito Y. (2006). Identification of DNMT1 (DNA methyltransferase 1) hypomorphs in somatic knockouts suggests an essential role for DNMT1 in cell survival. Proc. Natl. Acad. Sci. U. S. A. 103, 10314080–10314085. 10.1073/pnas.0604602103 PMC1599915 16963560

[B58] Endres M. Fan G. Meisel A. Dirnagl U. Jaenisch R. (2001). Effects of cerebral ischemia in mice lacking DNA methyltransferase 1 in post-mitotic neurons. Neuroreport 12, 123763–123766. 10.1097/00001756-200112040-00032 11726790

[B59] Endres M. Meisel A. Biniszkiewicz D. Namura S. Prass K. Ruscher K. (2000). DNA methyltransferase contributes to delayed ischemic brain injury. J. Neurosci. 20, 203175–203181. 10.1523/jneurosci.20-09-03175.2000 PMC6773114 10777781

[B60] Esvald E. E. Tuvikene J. Sirp A. Patil S. Bramham C. R. Timmusk T. (2020). CREB family transcription factors are major mediators of BDNF transcriptional autoregulation in cortical neurons. J. Neurosci. 40, 401405–401426. 10.1523/jneurosci.0367-19.2019 PMC7044735 31915257

[B61] Faggi L. Porrini V. Lanzillotta A. Benarese M. Mota M. Tsoukalas D. (2019). A polyphenol-enriched supplement exerts potent epigenetic-protective activity in a cell-based model of brain ischemia. Nutrients 11, 345. 10.3390/nu11020345 30736313 PMC6412333

[B62] Fang M. Chen D. Yang C. S. (2007). Dietary polyphenols may affect DNA methylation. J. Nutr. 137223s-228s, 223S–228S. 10.1093/jn/137.1.223S 17182830

[B63] Faraco G. Pancani T. Formentini L. Mascagni P. Fossati G. Leoni F. (2006). Pharmacological inhibition of histone deacetylases by suberoylanilide hydroxamic acid specifically alters gene expression and reduces ischemic injury in the mouse brain. Mol. Pharmacol. 70, 701876–701884. 10.1124/mol.106.027912 16946032

[B64] Fatemi M. Hermann A. Pradhan S. Jeltsch A. (2001). The activity of the murine DNA methyltransferase Dnmt1 is controlled by interaction of the catalytic domain with the N-terminal part of the enzyme leading to an allosteric activation of the enzyme after binding to methylated DNA. J. Mol. Biol. 309, 3091189–3091199. 10.1006/jmbi.2001.4709 11399088

[B65] Feng J. Zhou Y. Campbell S. L. Le T. Li E. Sweatt J. D. (2010). Dnmt1 and Dnmt3a maintain DNA methylation and regulate synaptic function in adult forebrain neurons. Nat. Neurosci. 13, 13423–13430. 10.1038/nn.2514 PMC3060772 20228804

[B66] Formisano L. Guida N. Valsecchi V. Cantile M. Cuomo O. Vinciguerra A. (2015). “Sp3/REST/HDAC1/HDAC2 complex represses and Sp1/HIF-1/p300 complex activates ncx1 gene transcription,” in Brain ischemia and in ischemic brain preconditioning, by epigenetic mechanism (J Neurosci.), 357332–357348. 10.1523/jneurosci.2174-14.2015 PMC6705442 25972164

[B67] Fu S. Kurzrock R. (2010). Development of curcumin as an epigenetic agent. Cancer 116, 1164670–1164676. 10.1002/cncr.25414 20597137

[B68] Gao J. Chen N. Li N. Xu F. Wang W. Lei Y. (2020). Neuroprotective effects of trilobatin, a novel naturally occurring Sirt3 agonist from Lithocarpus polystachyus rehd., mitigate cerebral ischemia/reperfusion injury: involvement of TLR4/NF-κB and nrf2/keap-1 signaling. Antioxid. Redox Signal 33, 33117–33143. 10.1089/ars.2019.7825 32212827

[B69] GaoJ.HeY.ShiF.HouF.WuX.YiY. (2025). Activation of Sirt6 by icariside Ⅱ alleviates depressive behaviors in mice with poststroke depression by modulating microbiota-gut-brain axis. J. Adv. Res. 10.1016/j.jare.2025.03.002 40037430

[B70] Gao X. Zeb S. He Y. Y. Guo Y. Zhu Y. M. Zhou X. Y. (2022). Valproic acid inhibits glial scar formation after ischemic stroke. Pharmacology 107, 107263–107280. 10.1159/000514951 35316816

[B71] GBD (2024). Global, regional, and national burden of disorders affecting the nervous system, 1990-2021: a systematic analysis for the Global Burden of Disease Study 2021. Lancet Neurol. 23, 23344–23381. 10.1016/s1474-4422(24)00038-3 PMC10949203 38493795

[B72] Goodman R. H. Smolik S. (2000). CBP/p300 in cell growth, transformation, and development. Genes Dev. 14, 141553–141577. 10.1101/gad.14.13.1553 10887150

[B73] Gou Y. Ye Q. Liang X. Zhang Q. Luo S. Liu H. (2021). Homocysteine restrains hippocampal neurogenesis in focal ischemic rat brain by inhibiting DNA methylation. Neurochem. Int. 147, 147105065. 10.1016/j.neuint.2021.105065 33940063

[B74] Guan J. S. Haggarty S. J. Giacometti E. Dannenberg J. H. Joseph N. Gao J. (2009). HDAC2 negatively regulates memory formation and synaptic plasticity. Nature 459, 45955–45960. 10.1038/nature07925 PMC3498958 19424149

[B75] Guida N. Serani A. Sanguigno L. Mascolo L. Cuomo O. Fioriniello S. (2024). Stroke causes DNA methylation at Ncx1 heart promoter in the brain via DNMT1/MeCP2/REST epigenetic complex. J. Am. Heart Assoc. 13, 13e030460. 10.1161/jaha.123.030460 PMC11010005 38456444

[B76] Guttzeit S. Backs J. (2022). Post-translational modifications talk and crosstalk to class IIa histone deacetylases. J. Mol. Cell Cardiol. 162, 16253–16261. 10.1016/j.yjmcc.2021.08.007 34416247

[B77] Haberland M. Montgomery R. L. Olson E. N. (2009). The many roles of histone deacetylases in development and physiology: implications for disease and therapy. Nat. Rev. Genet. 10, 1032–1042. 10.1038/nrg2485 PMC3215088 19065135

[B78] Han B. Wang M. Li J. Chen Q. Sun N. Yang X. (2023). Perspectives and new aspects of histone deacetylase inhibitors in the therapy of CNS diseases. Eur. J. Med. Chem. 258, 258115613. 10.1016/j.ejmech.2023.115613 37399711

[B79] Hatami M. Abdolahi M. Soveyd N. Djalali M. Togha M. Honarvar N. M. (2019). Molecular mechanisms of curcumin in neuroinflammatory disorders: a mini review of current evidences. Endocr. Metab. Immune Disord. Drug Targets 19, 19247–19258. 10.2174/1871530319666181129103056 30488803

[B80] He T. Shang J. Gao C. Guan X. Chen Y. Zhu L. (2021). A novel SIRT6 activator ameliorates neuroinflammation and ischemic brain injury via EZH2/FOXC1 axis. Acta Pharm. Sin. B 11, 11708–11726. 10.1016/j.apsb.2020.11.002 PMC7982432 33777677

[B81] He Y. F. Li B. Z. Li Z. Liu P. Wang Y. Tang Q. (2011). Tet-mediated formation of 5-carboxylcytosine and its excision by TDG in mammalian DNA. Science 333, 3331303–3331307. 10.1126/science.1210944 PMC3462231 21817016

[B82] Hebbes T. R. Thorne A. W. Crane-Robinson C. (1988). A direct link between core histone acetylation and transcriptionally active chromatin. Embo J. 7, 71395–71402. 10.1002/j.1460-2075.1988.tb02956.x PMC458389 3409869

[B83] Hernández-Jiménez M. Hurtado O. Cuartero M. I. Ballesteros I. Moraga A. Pradillo J. M. (2013). Silent information regulator 1 protects the brain against cerebral ischemic damage. Stroke, 442333–442337. 10.1161/strokeaha.113.001715 23723308

[B84] Hon G. C. Song C. X. Du T. Jin F. Selvaraj S. Lee A. Y. (2014). 5mC oxidation by Tet2 modulates enhancer activity and timing of transcriptome reprogramming during differentiation. Mol. Cell 56, 56286–56297. 10.1016/j.molcel.2014.08.026 PMC4319980 25263596

[B85] Hu J. Duan H. Zou J. Ding W. Wei Z. Peng Q. (2024). METTL3-dependent N6-methyladenosine modification is involved in berberine-mediated neuroprotection in ischemic stroke by enhancing the stability of NEAT1 in astrocytes. Aging 16, 16299–16321. 10.18632/aging.205369 PMC10817396 38180752

[B86] Hu J. X. Ma W. J. He L. Y. Zhang C. H. Zhang C. Wang Y. (2022). Macrophage migration inhibitory factor (MIF) acetylation protects neurons from ischemic injury. Cell Death Dis. 13466, 466. 10.1038/s41419-022-04918-2 PMC9117661 35585040

[B87] Huang F. Luo L. Wu Y. Xia D. Xu F. Gao J. (2022). Trilobatin promotes angiogenesis after cerebral ischemia-reperfusion injury via SIRT7/VEGFA signaling pathway in rats. Phytother. Res. 36, 362940–362951. 10.1002/ptr.7487 35537702

[B88] Huang Y. Chavez L. Chang X. Wang X. Pastor W. A. Kang J. (2014). Distinct roles of the methylcytosine oxidases Tet1 and Tet2 in mouse embryonic stem cells. Proc. Natl. Acad. Sci. U. S. A. 111, 1111361–1111366. 10.1073/pnas.1322921111 PMC3910590 24474761

[B89] Hutnick L. K. Golshani P. Namihira M. Xue Z. Matynia A. Yang X. W. (2009). DNA hypomethylation restricted to the murine forebrain induces cortical degeneration and impairs postnatal neuronal maturation. Hum. Mol. Genet. 18, 182875–182888. 10.1093/hmg/ddp222 PMC2706688 19433415

[B90] Hwang J. Y. Aromolaran K. A. Zukin R. S. (2017). The emerging field of epigenetics in neurodegeneration and neuroprotection. Nat. Rev. Neurosci. 18, 18347–18361. 10.1038/nrn.2017.46 PMC6380351 28515491

[B91] Inglis G. A. S. (2024). HDAC3 Stokes microglia in stroke. Nat. Neurosci. 27606, 606. 10.1038/s41593-024-01622-6 38589547

[B92] Ionescu V. S. Popa A. Alexandru A. Manole E. Neagu M. Pop S. (2021). Dietary phytoestrogens and their metabolites as epigenetic modulators with impact on human health. Antioxidants (Basel) 10, 1893. 10.3390/antiox10121893 34942997 PMC8750933

[B93] Ito S. D'Alessio A. C. Taranova O. V. Hong K. Sowers L. C. Zhang Y. (2010). Role of Tet proteins in 5mC to 5hmC conversion, ES-cell self-renewal and inner cell mass specification. Nature, 4661129–4661133. 10.1038/nature09303 PMC3491567 20639862

[B94] Ji F. Zhao C. Wang B. Tang Y. Miao Z. Wang Y. (2018). The role of 5-hydroxymethylcytosine in mitochondria after ischemic stroke. J. Neurosci. Res. 96, 961717–961726. 10.1002/jnr.24274 30043506

[B95] Jiao F. Wang Y. Zhang W. Zhang H. Chen Q. Wang L. (2020). AGK2 alleviates lipopolysaccharide induced neuroinflammation through regulation of mitogen-activated protein kinase phosphatase-1. J. Neuroimmune Pharmacol. 15, 15196–15208. 10.1007/s11481-019-09890-x 31786712

[B96] Jin S. G. Zhang Z. M. Dunwell T. L. Harter M. R. Wu X. Johnson J. (2016). Tet3 reads 5-carboxylcytosine through its CXXC domain and is a potential guardian against neurodegeneration. Cell Rep. 14, 14493–14505. 10.1016/j.celrep.2015.12.044 PMC4731272 26774490

[B97] Johnstone R. W. (2002). Histone-deacetylase inhibitors: novel drugs for the treatment of cancer. Nat. Rev. Drug Discov. 1, 1287–1299. 10.1038/nrd772 12120280

[B98] Joshi K. Zhang L. Breslin S. J. P. Kini A. R. Zhang J. (2022). Role of TET dioxygenases in the regulation of both normal and pathological hematopoiesis. J. Exp. Clin. Cancer Res., 41294. 10.1186/s13046-022-02496-x PMC9540719 36203205

[B99] Kabir F. Atkinson R. Cook A. L. Phipps A. J. King A. E. (2022). The role of altered protein acetylation in neurodegenerative disease. Front. Aging Neurosci. 14, 141025473. 10.3389/fnagi.2022.1025473 PMC9845957 36688174

[B100] Kalaiarasi A. Anusha C. Sankar R. Rajasekaran S. John Marshal J. Muthusamy K. (2016). Plant isoquinoline alkaloid berberine exhibits chromatin remodeling by modulation of histone deacetylase to induce growth arrest and apoptosis in the A549 cell line. J. Agric. Food Chem. 64, 649542–649550. 10.1021/acs.jafc.6b04453 27936791

[B101] Kalani A. Kamat P. K. Tyagi N. (2015). Diabetic stroke severity: epigenetic remodeling and neuronal, glial, and vascular dysfunction. Diabetes 64, 644260–644271. 10.2337/db15-0422 PMC4876759 26470785

[B102] Kang S. K. Lee D. H. Bae Y. C. Kim H. K. Baik S. Y. Jung J. S. (2003). Improvement of neurological deficits by intracerebral transplantation of human adipose tissue-derived stromal cells after cerebral ischemia in rats. Exp. Neurol. 183, 183355–183366. 10.1016/s0014-4886(03)00089-x 14552877

[B103] Kang Y. Sun Y. Li T. Ren Z. (2020). Garcinol protects against cerebral ischemia-reperfusion injury *in vivo* and *in vitro* by inhibiting inflammation and oxidative stress. Mol. Cell Probes 54, 54101672. 10.1016/j.mcp.2020.101672 33186709

[B104] Karnay A. Karisetty B. C. Beaver M. Elefant F. (2019). Hippocampal stimulation promotes intracellular Tip60 dynamics with concomitant genome reorganization and synaptic gene activation. Mol. Cell Neurosci. 101, 101103412. 10.1016/j.mcn.2019.103412 PMC6879871 31682915

[B105] Kassis H. Shehadah A. Chopp M. Roberts C. Zhang Z. G. (2015). Stroke induces nuclear shuttling of histone deacetylase 4. Stroke 46, 461909–461915. 10.1161/strokeaha.115.009046 PMC4480163 25967576

[B106] Kazantsev A. G. Thompson L. M. (2008). Therapeutic application of histone deacetylase inhibitors for central nervous system disorders. Nat. Rev. Drug Discov. 7, 7854–7868. 10.1038/nrd2681 18827828

[B107] Khoury N. Koronowski K. B. Perez-Pinzon M. A. (2016). Long-term window of ischemic tolerance: an evolutionarily conserved form of metabolic plasticity regulated by epigenetic modifications? J. Neurol. Neuromedicine 1, 16–12. 10.29245/2572.942x/2016/2.1021 PMC5081687 27796011

[B108] Khoury N. Xu J. Stegelmann S. D. Jackson C. W. Koronowski K. B. Dave K. R. (2019). Resveratrol preconditioning induces genomic and metabolic adaptations within the long-term window of cerebral ischemic tolerance leading to bioenergetic efficiency. Mol. Neurobiol. 56, 564549–564565. 10.1007/s12035-018-1380-6 PMC6475499 30343466

[B109] Kim H. J. Rowe M. Ren M. Hong J. S. Chen P. S. Chuang D. M. (2007). Histone deacetylase inhibitors exhibit anti-inflammatory and neuroprotective effects in a rat permanent ischemic model of stroke: multiple mechanisms of action. J. Pharmacol. Exp. Ther. 321, 321892–321901. 10.1124/jpet.107.120188 17371805

[B110] Kim J. Y. Shen S. Dietz K. He Y. Howell O. Reynolds R. (2010). HDAC1 nuclear export induced by pathological conditions is essential for the onset of axonal damage. Nat. Neurosci. 13, 13180–13189. 10.1038/nn.2471 PMC2829989 20037577

[B111] Kuo T. T. Wang V. Wu J. S. Chen Y. H. Tseng K. Y. (2021). Post-stroke delivery of valproic acid promotes functional recovery and differentially modifies responses of peri-infarct microglia. Front. Mol. Neurosci. 14, 14639145. 10.3389/fnmol.2021.639145 PMC8194695 34122007

[B112] Lahm A. Paolini C. Pallaoro M. Nardi M. C. Jones P. Neddermann P. (2007). Unraveling the hidden catalytic activity of vertebrate class IIa histone deacetylases. Proc. Natl. Acad. Sci. U. S. A. 104, 10417335–10417340. 10.1073/pnas.0706487104 PMC2077257 17956988

[B113] Lanzillotta A. Pignataro G. Branca C. Cuomo O. Sarnico I. Benarese M. (2013). Targeted acetylation of NF-kappaB/RelA and histones by epigenetic drugs reduces post-ischemic brain injury in mice with an extended therapeutic window. Neurobiol. Dis. 49, 49177–49189. 10.1016/j.nbd.2012.08.018 22971966

[B114] Laranjeira A. B. A. Hollingshead M. G. Nguyen D. Kinders R. J. Doroshow J. H. Yang S. X. (2023). DNA damage, demethylation and anticancer activity of DNA methyltransferase (DNMT) inhibitors. Sci. Rep. 13, 135964. 10.1038/s41598-023-32509-4 PMC10097729 37045940

[B115] LaSalle J. M. (2025). DNA methylation biomarkers of intellectual/developmental disability across the lifespan. J. Neurodev. Disord. 1710, 10. 10.1186/s11689-025-09598-5 PMC11841270 39972408

[B116] Lasko L. M. Jakob C. G. Edalji R. P. Qiu W. Montgomery D. Digiammarino E. L. (2017). Discovery of a selective catalytic p300/CBP inhibitor that targets lineage-specific tumours. Nature 550, 550128–550132. 10.1038/nature24028 PMC6050590 28953875

[B117] Lau O. D. Kundu T. K. Soccio R. E. Ait-Si-Ali S. Khalil E. M. Vassilev A. (2000). HATs off: selective synthetic inhibitors of the histone acetyltransferases p300 and PCAF. Mol. Cell 5, 5589–5595. 10.1016/s1097-2765(00)80452-9 10882143

[B118] Lee J. C. Park J. H. Yan B. C. Kim I. H. Cho G. S. Jeoung D. (2013). Effects of transient cerebral ischemia on the expression of DNA methyltransferase 1 in the gerbil hippocampal CA1 region. Neurochem. Res. 38, 3874–3881. 10.1007/s11064-012-0890-2 22987057

[B119] Lee S. Lee B. Lee J. W. Lee S. K. (2009). Retinoid signaling and neurogenin2 function are coupled for the specification of spinal motor neurons through a chromatin modifier CBP. Neuron 62, 62641–62654. 10.1016/j.neuron.2009.04.025 PMC2705669 19524524

[B120] Li J. Wei G. Song Z. Chen Z. Gu J. Zhang L. (2024). SIRT5 regulates ferroptosis through the Nrf2/HO-1 signaling Axis to participate in ischemia-reperfusion injury in ischemic stroke. Neurochem. Res. 49, 49998–51007. 10.1007/s11064-023-04095-4 38170384

[B121] Li M. Li S. C. Dou B. K. Zou Y. X. Han H. Z. Liu D. X. (2020). Cycloastragenol upregulates SIRT1 expression, attenuates apoptosis and suppresses neuroinflammation after brain ischemia. Acta Pharmacol. Sin. 41, 411025–411032. 10.1038/s41401-020-0386-6 PMC7471431 32203080

[B122] Li N. Yuan Q. Cao X. L. Zhang Y. Min Z. L. Xu S. Q. (2017). Opposite effects of HDAC5 and p300 on MRTF-A-related neuronal apoptosis during ischemia/reperfusion injury in rats. Cell Death Dis. 8, 8e2624. 10.1038/cddis.2017.16 PMC5386465 28230854

[B123] Li Q. Zhang C. Sun X. Wang M. Zhang Z. Chen R. (2024). Forsythoside B alleviates cerebral ischemia-reperfusion injury via inhibiting NLRP3 inflammasome mediated by SIRT1 activation. PLoS One 19, 19e0305541. 10.1371/journal.pone.0305541 PMC11182500 38885233

[B124] Li T. T. Zhao D. M. Wei Y. T. Li J. B. Li X. F. Wan Q. (2023). Effect and mechanism of sodium butyrate on neuronal recovery and prognosis in diabetic stroke. J. Neuroimmune Pharmacol. 18, 18366–18382. 10.1007/s11481-023-10071-0 37318680

[B125] Li X. Sui Y. (2020). Valproate improves middle cerebral artery occlusion-induced ischemic cerebral disorders in mice and oxygen-glucose deprivation-induced injuries in microglia by modulating RMRP/PI3K/Akt axis. Brain Res. 1747, 1747147039. 10.1016/j.brainres.2020.147039 32745656

[B126] Li Y. Ma Y. Song L. Yu L. Zhang L. Zhang Y. (2018). SIRT3 deficiency exacerbates p53/Parkin-mediated mitophagy inhibition and promotes mitochondrial dysfunction: implication for aged hearts. Int. J. Mol. Med. 41, 413517–413526. 10.3892/ijmm.2018.3555 29532856

[B127] Liao H. Huang J. Liu J. Zhu H. Chen Y. Li X. (2023). Sirt1 regulates microglial activation and inflammation following oxygen-glucose deprivation/reoxygenation injury by targeting the Shh/Gli-1 signaling pathway. Mol. Biol. Rep. 50, 503317–503327. 10.1007/s11033-022-08167-6 PMC10042964 36725745

[B128] Liao J. Karnik R. Gu H. Ziller M. J. Clement K. Tsankov A. M. (2015). Targeted disruption of DNMT1, DNMT3A and DNMT3B in human embryonic stem cells. Nat. Genet. 47, 47469–47478. 10.1038/ng.3258 PMC4414868 25822089

[B129] Liberale L. Gaul D. S. Akhmedov A. Bonetti N. R. Nageswaran V. Costantino S. (2020). Endothelial SIRT6 blunts stroke size and neurological deficit by preserving blood-brain barrier integrity: a translational study. Eur. Heart J. 41, 411575–411587. 10.1093/eurheartj/ehz712 31603194

[B130] Lin H. Bei Y. Shen Z. Wei T. Ge Y. Yu L. (2024). HDAC9 deficiency upregulates cGMP-dependent kinase II to mitigate neuronal apoptosis in ischemic stroke. Transl. Stroke Res. 10.1007/s12975-024-01272-7 38940872

[B131] Lin Y. Liu M. Deng P. Zhang J. (2024). TET1 mediated m5C modification of RelB aggravates cerebral ischemia/reperfusion-induced neuroinflammation through regulating microglia polarization. Cell Signal 120, 120111210. 10.1016/j.cellsig.2024.111210 38705503

[B132] Lin Y. H. Dong J. Tang Y. Ni H. Y. Zhang Y. Su P. (2017). Opening a new time window for treatment of stroke by targeting HDAC2. J. Neurosci. 37, 376712–376728. 10.1523/jneurosci.0341-17.2017 PMC6596556 28592694

[B133] Lingling D. Miaomiao Q. Yili L. Hongyun H. Yihao D. (2022). Attenuation of histone H4 lysine 16 acetylation (H4K16ac) elicits a neuroprotection against ischemic stroke by alleviating the autophagic/lysosomal dysfunction in neurons at the penumbra. Brain Res. Bull. 184, 18424–18433. 10.1016/j.brainresbull.2022.03.013 35351588

[B134] Liu D. Li G. Zuo Y. (2019). Function determinants of TET proteins: the arrangements of sequence motifs with specific codes. Brief. Bioinform 20, 201826–201835. 10.1093/bib/bby053 29947743

[B135] Liu J. W. Zhang Z. Q. Zhu Z. C. Li K. Xu Q. Zhang J. (2024). Loss of TET activity in the postnatal mouse brain perturbs synaptic gene expression and impairs cognitive function. Neurosci. Bull. 40, 401699–401712. 10.1007/s12264-024-01302-2 PMC11607366 39395911

[B136] Liu S. S. Wu F. Jin Y. M. Chang W. Q. Xu T. M. (2020). HDAC11: a rising star in epigenetics. Biomed. Pharmacother. 131, 131110607. 10.1016/j.biopha.2020.110607 32841898

[B137] Liu X. Khan A. Li H. Wang S. Chen X. Huang H. (2022). Ascorbic acid in epigenetic reprogramming. Curr. Stem Cell Res. Ther. 1713-25, 13–25. 10.2174/1574888x16666210714152730 34264189

[B138] Liu Y. Wang L. Yang G. Chi X. Liang X. Zhang Y. (2023). Sirtuins: promising therapeutic targets to treat ischemic stroke. Biomolecules 13, 1210. 10.3390/biom13081210 37627275 PMC10452362

[B139] Liu Y. L. Yang H. P. Gong L. Tang C. L. Wang H. J. (2011a). Hypomethylation effects of curcumin, demethoxycurcumin and bisdemethoxycurcumin on WIF-1 promoter in non-small cell lung cancer cell lines. Mol. Med. Rep. 4, 4675–4679. 10.3892/mmr.2011.473 21484077

[B140] Liu Y. L. Yang H. P. Zhou X. D. Gong L. Tang C. L. Wang H. J. (2011b). The hypomethylation agent bisdemethoxycurcumin acts on the WIF-1 promoter, inhibits the canonical Wnt pathway and induces apoptosis in human non-small-cell lung cancer. Curr. Cancer Drug Targets 11, 111098–111110. 10.2174/156800911798073041 21933103

[B141] Liu Z. Xie Z. Jones W. Pavlovicz R. E. Liu S. Yu J. (2009). Curcumin is a potent DNA hypomethylation agent. Bioorg Med. Chem. Lett. 19, 19706–19709. 10.1016/j.bmcl.2008.12.041 19112019

[B142] Lorsbach R. B. Moore J. Mathew S. Raimondi S. C. Mukatira S. T. Downing J. R. (2003). TET1, a member of a novel protein family, is fused to MLL in acute myeloid leukemia containing the t(10;11)(q22;q23). Leukemia 17, 17637–17641. 10.1038/sj.leu.2402834 12646957

[B143] Lu F. Liu Y. Jiang L. Yamaguchi S. Zhang Y. (2014). Role of Tet proteins in enhancer activity and telomere elongation. Genes Dev. 28, 282103–282119. 10.1101/gad.248005.114 PMC4180973 25223896

[B144] Lu Y. Chen Y. Xu S. Wei L. Zhang Y. Chen W. (2023). HDAC inhibitor attenuates rat traumatic brain injury induced neurological impairments. Heliyon. 9e18485. 9, e18485. 10.1016/j.heliyon.2023.e18485 PMC10407045 37560709

[B145] Lv J. Tian J. Zheng G. Zhao J. (2017). Sirtuin7 is involved in protecting neurons against oxygen-glucose deprivation and reoxygenation-induced injury through regulation of the p53 signaling pathway. J. Biochem. Mol. Toxicol. 31. 10.1002/jbt.21955 28675767

[B146] Lyu T. J. Qiu X. Wang Y. Zhang L. Dai Y. Wang X. (2024). DNMT3A dysfunction promotes neuroinflammation and exacerbates acute ischemic stroke. MedComm 5, 5e652. 10.1002/mco2.652 PMC11246610 39006763

[B147] Ma Q. Dasgupta C. Shen G. Li Y. Zhang L. (2021). MicroRNA-210 downregulates TET2 and contributes to inflammatory response in neonatal hypoxic-ischemic brain injury. J. Neuroinflammation 186, 6. 10.1186/s12974-020-02068-w PMC7786974 33402183

[B148] MacArthur I. C. Ma L. Huang C. Y. Bhavsar H. Suzuki M. Dawlaty M. M. (2024). Developmental DNA demethylation is a determinant of neural stem cell identity and gliogenic competence. Sci. Adv. 10, 10eado5424. 10.1126/sciadv.ado5424 PMC11352921 39196941

[B149] Marek R. Coelho C. M. Sullivan R. K. Baker-Andresen D. Li X. Ratnu V. (2011). Paradoxical enhancement of fear extinction memory and synaptic plasticity by inhibition of the histone acetyltransferase p300. J. Neurosci. 31, 317486–317491. 10.1523/jneurosci.0133-11.2011 PMC3458795 21593332

[B150] Markus H. S. (2023). HDAC9 inhibition as a novel treatment for stroke. Stroke 54, 543182–543189. 10.1161/strokeaha.123.044862 37942644

[B151] Marmorstein R. Zhou M. M. (2014). Writers and readers of histone acetylation: structure, mechanism, and inhibition. Cold Spring Harb. Perspect. Biol. 6a018762, a018762. 10.1101/cshperspect.a018762 PMC4067988 24984779

[B152] Martin S. S. Aday A. W. Almarzooq Z. I. Anderson C. A. M. Arora P. Avery C. L. (2024). 2024 heart disease and stroke statistics: a report of us and global data from the American heart association. Circulation 149, e347–e913. 149e347-e913. 10.1161/cir.0000000000001209 38264914 PMC12146881

[B153] Martino S. Gargano S. Carollo P. S. Di Leonardo A. Barra V. (2024). DNMT1 prolonged absence is a tunable cellular stress that triggers cell proliferation arrest to protect from major DNA methylation loss. Cell Mol. Life Sci. 827, 7. 10.1007/s00018-024-05547-y PMC11655745 39694934

[B154] Maugeri A. Barchitta M. Mazzone M. G. Giuliano F. Basile G. Agodi A. (2018). Resveratrol modulates SIRT1 and DNMT functions and restores LINE-1 methylation levels in ARPE-19 cells under oxidative stress and inflammation. Int. J. Mol. Sci. 19, 2118. 10.3390/ijms19072118 30037017 PMC6073744

[B155] Miao Y. Zhao S. Gao Y. Wang R. Wu Q. Wu H. (2016). Curcumin pretreatment attenuates inflammation and mitochondrial dysfunction in experimental stroke: the possible role of Sirt1 signaling. Brain Res. Bull. 121, 1219–1315. 10.1016/j.brainresbull.2015.11.019 26639783

[B156] Miao Z. He Y. Xin N. Sun M. Chen L. Lin L. (2015). Altering 5-hydroxymethylcytosine modification impacts ischemic brain injury. Hum. Mol. Genet. 24, 245855–245866. 10.1093/hmg/ddv307 PMC4581609 26231219

[B157] Milazzo G. Mercatelli D. Di Muzio G. Triboli L. De Rosa P. Perini G. (2020). Histone deacetylases (HDACs): evolution, specificity, role in transcriptional complexes, and pharmacological actionability. Genes (Basel) 11, 556. 10.3390/genes11050556 32429325 PMC7288346

[B158] Mondal N. K. Behera J. Kelly K. E. George A. K. Tyagi P. K. Tyagi N. (2019). Tetrahydrocurcumin epigenetically mitigates mitochondrial dysfunction in brain vasculature during ischemic stroke. Neurochem. Int. 122, 122120–122138. 10.1016/j.neuint.2018.11.015 PMC6666268 30472160

[B159] Monsour M. Gordon J. Lockard G. Alayli A. Elsayed B. Connolly J. (2022). Minor changes for a major impact: a review of epigenetic modifications in cell-based therapies for stroke. Int. J. Mol. Sci. 23, 13106. 10.3390/ijms232113106 36361891 PMC9656972

[B160] Morris-Blanco K. C. Chokkalla A. K. Bertogliat M. J. Vemuganti R. (2021). TET3 regulates DNA hydroxymethylation of neuroprotective genes following focal ischemia. J. Cereb. Blood Flow. Metab. 41, 41590–41603. 10.1177/0271678x20912965 PMC7922754 32380888

[B161] Morris-Blanco K. C. Chokkalla A. K. Kim T. Bhatula S. Bertogliat M. J. Gaillard A. B. (2022). High-dose vitamin C prevents secondary brain damage after stroke via epigenetic reprogramming of neuroprotective genes. Transl. Stroke Res. 13, 131017–131036. 10.1007/s12975-022-01007-6 PMC9485293 35306630

[B162] Morris-Blanco K. C. Dave K. R. Saul I. Koronowski K. B. Stradecki H. M. Perez-Pinzon M. A. (2016). Protein kinase C epsilon promotes cerebral ischemic tolerance via modulation of mitochondrial Sirt5. Sci. Rep. 6, 629790. 10.1038/srep29790 PMC4951704 27435822

[B163] Nandakumar V. Vaid M. Katiyar S. K. (2011). (-)-Epigallocatechin-3-gallate reactivates silenced tumor suppressor genes, Cip1/p21 and p16INK4a, by reducing DNA methylation and increasing histones acetylation in human skin cancer cells. Carcinogenesis 32, 32537–32544. 10.1093/carcin/bgq285 PMC3066414 21209038

[B164] Noguchi H. Murao N. Kimura A. Matsuda T. Namihira M. Nakashima K. (2016). DNA methyltransferase 1 is indispensable for development of the hippocampal dentate gyrus. J. Neurosci. 36, 366050–366068. 10.1523/jneurosci.0512-16.2016 PMC6601819 27251626

[B165] Okano M. Bell D. W. Haber D. A. Li E. (1999). DNA methyltransferases Dnmt3a and Dnmt3b are essential for *de novo* methylation and mammalian development. Cell 99, 99247–99257. 10.1016/s0092-8674(00)81656-6 10555141

[B166] Ooi S. K. Qiu C. Bernstein E. Li K. Jia D. Yang Z. (2007). DNMT3L connects unmethylated lysine 4 of histone H3 to *de novo* methylation of DNA. Nature 448, 448714–448717. 10.1038/nature05987 PMC2650820 17687327

[B167] Ortega M. A. De Leon-Oliva D. Garcia-Montero C. Fraile-Martinez O. Boaru D. L. Del Val Toledo Lobo M. (2023). Understanding HAT1: a comprehensive review of noncanonical roles and connection with disease. Genes (Basel) 14, 915. 10.3390/genes14040915 37107673 PMC10137880

[B168] Oshikawa M. Okada K. Tabata H. Nagata K. I. Ajioka I. (2017). Dnmt1-dependent Chk1 pathway suppression is protective against neuron division. Development 144, 1443303–1443314. 10.1242/dev.154013 28928282

[B169] Ou Z. Wang Y. Yao J. Chen L. Miao H. Han Y. (2023). Astragaloside IV promotes angiogenesis by targeting SIRT7/VEGFA signaling pathway to improve brain injury after cerebral infarction in rats. Biomed. Pharmacother. 168, 168115598. 10.1016/j.biopha.2023.115598 37820565

[B170] Owjfard M. Rahimian Z. Karimi F. Borhani-Haghighi A. Mallahzadeh A. (2024). A comprehensive review on the neuroprotective potential of resveratrol in ischemic stroke. Heliyon 10e34121, e34121. 10.1016/j.heliyon.2024.e34121 PMC11284444 39082038

[B171] Park J. M. Kim T. H. Jo S. H. Kim M. Y. Ahn Y. H. (2015). Acetylation of glucokinase regulatory protein decreases glucose metabolism by suppressing glucokinase activity. Sci. Rep. 5, 517395. 10.1038/srep17395 PMC4664969 26620281

[B172] Park M. J. Sohrabji F. (2016). The histone deacetylase inhibitor, sodium butyrate, exhibits neuroprotective effects for ischemic stroke in middle-aged female rats. J. Neuroinflammation 13300, 300. 10.1186/s12974-016-0765-6 PMC5131416 27905989

[B173] Park S. J. Ahmad F. Philp A. Baar K. Williams T. Luo H. (2012). Resveratrol ameliorates aging-related metabolic phenotypes by inhibiting cAMP phosphodiesterases. Cell 148, 148421–148433. 10.1016/j.cell.2012.01.017 PMC3431801 22304913

[B174] Park S. Y. Kim J. S. (2020). A short guide to histone deacetylases including recent progress on class II enzymes. Exp. Mol. Med. 52, 52204–52212. 10.1038/s12276-020-0382-4 PMC7062823 32071378

[B175] Patnala R. Arumugam T. V. Gupta N. Dheen S. T. (2017). HDAC inhibitor sodium butyrate-mediated epigenetic regulation enhances neuroprotective function of microglia during ischemic stroke. Mol. Neurobiol. 54, 546391–546411. 10.1007/s12035-016-0149-z 27722928

[B176] Pensold D. Reichard J. Van Loo K. M. J. Ciganok N. Hahn A. Bayer C. (2020). DNA methylation-mediated modulation of endocytosis as potential mechanism for synaptic function regulation in murine inhibitory cortical interneurons. Cereb. Cortex 30, 303921–303937. 10.1093/cercor/bhaa009 PMC7264686 32147726

[B177] Powers W. J. Rabinstein A. A. Ackerson T. Adeoye O. M. Bambakidis N. C. Becker K. (2019). Guidelines for the early management of patients with acute ischemic stroke: 2019 update to the 2018 guidelines for the early management of acute ischemic stroke: a guideline for healthcare professionals from the American heart association/American stroke association. Stroke 50, 50e344–e418. 10.1161/str.0000000000000211 31662037

[B178] Pratt K. J. B. Shea J. M. Remesal-Gomez L. Bieri G. Smith L. K. Couthouis J. (2023). Loss of neuronal Tet2 enhances hippocampal-dependent cognitive function. Cell Rep. 42, 42111926. 10.1016/j.celrep.2022.111926 36719799

[B179] Qi R. Zhang X. Xie Y. Jiang S. Liu Y. Liu X. (2019). 5-Aza-2'-deoxycytidine increases hypoxia tolerance-dependent autophagy in mouse neuronal cells by initiating the TSC1/mTOR pathway. Biomed. Pharmacother. 118, 118109219. 10.1016/j.biopha.2019.109219 31325707

[B180] Qiu Y. Xu Q. Xie P. He C. Li Q. Yao X. (2025). Epigenetic modifications and emerging therapeutic targets in cardiovascular aging and diseases. Pharmacol. Res. 211, 211107546. 10.1016/j.phrs.2024.107546 39674563

[B181] Ramadori G. Lee C. E. Bookout A. L. Lee S. Williams K. W. Anderson J. (2008). Brain SIRT1: anatomical distribution and regulation by energy availability. J. Neurosci. 289989-9996, 9989–9996. 10.1523/jneurosci.3257-08.2008 PMC2578850 18829956

[B182] Rathore A. S. Birla H. Singh S. S. Zahra W. Dilnashin H. Singh R. (2021). Epigenetic modulation in Parkinson's disease and potential treatment therapies. Neurochem. Res. 46, 461618–461626. 10.1007/s11064-021-03334-w 33900517

[B183] Raval A. P. Dave K. R. Pérez-Pinzón M. A. (2006). Resveratrol mimics ischemic preconditioning in the brain. J. Cereb. Blood Flow. Metab., 261141–261147. 10.1038/sj.jcbfm.9600262 16395277

[B184] Raz L. Zhang Q. G. Han D. Dong Y. De Sevilla L. Brann D. W. (2011). Acetylation of the pro-apoptotic factor, p53 in the hippocampus following cerebral ischemia and modulation by estrogen. PLoS One 6, 6e27039. 10.1371/journal.pone.0027039 PMC3202599 22046440

[B185] Ryu J. Y. Cerecedo-Lopez C. Yang H. Ryu I. Du R. (2024). Brain-targeted intranasal delivery of protein-based gene therapy for treatment of ischemic stroke. Theranostics 14, 144773–144786. 10.7150/thno.98088 PMC11373627 39239521

[B186] Sahafnejad Z. Ramazi S. Allahverdi A. (2023). An update of epigenetic drugs for the treatment of cancers and brain diseases: a comprehensive review. Genes (Basel) 14, 873. 10.3390/genes14040873 37107631 PMC10137918

[B187] Sang C. Li X. Liu J. Chen Z. Xia M. Yu M. (2024). Reversible acetylation of HDAC8 regulates cell cycle. EMBO Rep. 25, 253925–253943. 10.1038/s44319-024-00210-w PMC11387496 39043961

[B188] Santiago M. Antunes C. Guedes M. Iacovino M. Kyba M. Reik W. (2020). Tet3 regulates cellular identity and DNA methylation in neural progenitor cells. Cell Mol. Life Sci. 77, 772871–772883. 10.1007/s00018-019-03335-7 PMC7326798 31646359

[B189] Satoh A. Imai S. (2014). Systemic regulation of mammalian ageing and longevity by brain sirtuins. Nat. Commun. 54211, 4211. 10.1038/ncomms5211 PMC4521907 24967620

[B190] Sharifulina S. Dzreyan V. Guzenko V. Demyanenko S. (2021). Histone methyltransferases SUV39H1 and G9a and DNA methyltransferase DNMT1 in penumbra neurons and astrocytes after photothrombotic stroke. Int. J. Mol. Sci. 22, 12483. 10.3390/ijms222212483 34830365 PMC8619375

[B191] She D. T. Wong L. J. Baik S. H. Arumugam T. V. (2018). SIRT2 inhibition confers neuroprotection by downregulation of FOXO3a and MAPK signaling pathways in ischemic stroke. Mol. Neurobiol. 55, 559188–559203. 10.1007/s12035-018-1058-0 29654491

[B192] Shi G. Feng J. Jian L. Y. Fan X. Y. (2023). DNA hypomethylation promotes learning and memory recovery in a rat model of cerebral ischemia/reperfusion injury. Neural Regen. Res. 18, 18863–18868. 10.4103/1673-5374.353494 PMC9700107 36204855

[B193] Shi W. Wei X. Wang Z. Han H. Fu Y. Liu J. (2016). HDAC9 exacerbates endothelial injury in cerebral ischaemia/reperfusion injury. J. Cell Mol. Med. 20, 201139–201149. 10.1111/jcmm.12803 PMC4882992 26865248

[B194] Shi Y. H. Zhang X. L. Ying P. J. Wu Z. Q. Lin L. L. Chen W. (2021). Neuroprotective effect of astragaloside IV on cerebral ischemia/reperfusion injury rats through sirt1/mapt pathway. Front. Pharmacol. 12, 12639898. 10.3389/fphar.2021.639898 PMC8033022 33841157

[B195] Shin J. A. Lee K. E. Kim H. S. Park E. M. (2012). Acute resveratrol treatment modulates multiple signaling pathways in the ischemic brain. Neurochem. Res. 37, 372686–372696. 10.1007/s11064-012-0858-2 22878646

[B196] Shu L. Xu C. Q. Yan Z. Y. Yan Y. Jiang S. Z. Wang Y. R. (2019). Post-stroke microglia induce Sirtuin2 expression to suppress the anti-inflammatory function of infiltrating regulatory T cells. Inflammation 42, 421968–421979. 10.1007/s10753-019-01057-3 31297748

[B197] Singh A. K. Joshi I. Reddy N. M. N. Purushotham S. S. Eswaramoorthy M. Vasudevan M. (2025). Epigenetic modulation rescues neurodevelopmental deficits in Syngap1(+/-) mice. Aging Cell 24, e14408. 10.1111/acel.14408 39878322 PMC11896221

[B198] Singh D. Khan M. A. Siddique H. R. (2023). Role of epigenetic drugs in sensitizing cancers to anticancer therapies: emerging trends and clinical advancements. Epigenomics 15, 15517–15537. 10.2217/epi-2023-0142 37313832

[B199] Singh P. Thakur M. K. (2014). Reduced recognition memory is correlated with decrease in DNA methyltransferase1 and increase in histone deacetylase2 protein expression in old male mice. Biogerontology 15, 15339–15346. 10.1007/s10522-014-9504-5 24924148

[B200] Smith Z. D. Meissner A. (2013). DNA methylation: roles in mammalian development. Nat. Rev. Genet. 14, 14204–14220. 10.1038/nrg3354 23400093

[B201] Song J. He K. Yang L. Shen J. (2022). Sevoflurane protects mice from cerebral ischaemic injury by regulating microRNA-203-3p/HDAC4/Bcl-2 axis. Eur. J. Neurosci. 55, 551695–551708. 10.1111/ejn.15622 35141965

[B202] Song M. Yi F. Zeng F. Zheng L. Huang L. Sun X. (2024). USP18 stabilized FTO protein to activate mitophagy in ischemic stroke through repressing m6A modification of SIRT6. Mol. Neurobiol. 61, 616658–616674. 10.1007/s12035-024-04001-1 38340205

[B203] Stanzione R. Cotugno M. Bianchi F. Marchitti S. Forte M. Volpe M. (2020). Pathogenesis of ischemic stroke: role of epigenetic mechanisms. Genes (Basel) 11, 89. 10.3390/genes11010089 31941075 PMC7017187

[B204] Stoyanova E. Riad M. Rao A. Heintz N. (2021). 5-Hydroxymethylcytosine-mediated active demethylation is required for mammalian neuronal differentiation and function. Elife 10, e66973. 10.7554/eLife.66973 34919053 PMC8683082

[B205] Suda S. Katsura K. Kanamaru T. Saito M. Katayama Y. (2013). Valproic acid attenuates ischemia-reperfusion injury in the rat brain through inhibition of oxidative stress and inflammation. Eur. J. Pharmacol. 707, 70726–70731. 10.1016/j.ejphar.2013.03.020 23541723

[B206] Sugo N. Oshiro H. Takemura M. Kobayashi T. Kohno Y. Uesaka N. (2010). Nucleocytoplasmic translocation of HDAC9 regulates gene expression and dendritic growth in developing cortical neurons. Eur. J. Neurosci. 31, 311521–311532. 10.1111/j.1460-9568.2010.07218.x 20525066

[B207] Tan Y. Wang Z. Liu T. Gao P. Xu S. Tan L. (2022). RNA interference-mediated silencing of DNA methyltransferase 1 attenuates neuropathic pain by accelerating microglia M2 polarization. BMC Neurol. 22376, 376. 10.1186/s12883-022-02860-6 PMC9526327 36183073

[B208] Tan Z. Dong F. Wu L. Xu G. Zhang F. (2024). Transcutaneous electrical acupoint stimulation attenuated neuroinflammation and oxidative stress by activating SIRT1-induced signaling pathway in MCAO/R rat models. Exp. Neurol. 373, 373114658. 10.1016/j.expneurol.2023.114658 38141805

[B209] Tanaka Y. Naruse I. Hongo T. Xu M. Nakahata T. Maekawa T. (2000). Extensive brain hemorrhage and embryonic lethality in a mouse null mutant of CREB-binding protein. Mech. Dev. 95, 95133–95145. 10.1016/s0925-4773(00)00360-9 10906457

[B210] Tang F. Guo S. Liao H. Yu P. Wang L. Song X. (2017). Resveratrol enhances neurite outgrowth and synaptogenesis via sonic hedgehog signaling following oxygen-glucose deprivation/reoxygenation injury. Cell Physiol. Biochem. 43, 43852–43869. 10.1159/000481611 28957797

[B211] Tang Y. Lin Y. H. Ni H. Y. Dong J. Yuan H. J. Zhang Y. (2017). Inhibiting histone deacetylase 2 (HDAC2) promotes functional recovery from stroke. J. Am. Heart Assoc. 6, e007236. 10.1161/jaha.117.007236 28982677 PMC5721897

[B212] Tanno M. Sakamoto J. Miura T. Shimamoto K. Horio Y. (2007). Nucleocytoplasmic shuttling of the NAD+-dependent histone deacetylase SIRT1. J. Biol. Chem. 282, 2826823–2826832. 10.1074/jbc.M609554200 17197703

[B213] Tao R. Coleman M. C. Pennington J. D. Ozden O. Park S. H. Jiang H. (2010). Sirt3-mediated deacetylation of evolutionarily conserved lysine 122 regulates MnSOD activity in response to stress. Mol. Cell 40, 40893–40904. 10.1016/j.molcel.2010.12.013 PMC3266626 21172655

[B214] Thangapandian S. John S. Lee Y. Arulalapperumal V. Lee K. W. (2012). Molecular modeling study on tunnel behavior in different histone deacetylase isoforms. PLoS One 7, 7e49327. 10.1371/journal.pone.0049327 PMC3510210 23209570

[B215] The Lancet (2024). A real chance to reduce death and disability from stroke. Lancet Neurol. 23749. 10.1016/s1474-4422(24)00283-7 39030027

[B216] Tian H. Luan P. Liu Y. Li G. (2024). Tet-mediated DNA methylation dynamics affect chromosome organization. Nucleic Acids Res. 52, 523654–523666. 10.1093/nar/gkae054 PMC11039979 38300758

[B217] Toth M. (2021). Epigenetic neuropharmacology: drugs affecting the epigenome in the brain. Annu. Rev. Pharmacol. Toxicol. 61, 61181–61201. 10.1146/annurev-pharmtox-030220-022920 PMC9915026 32997604

[B218] Trowbridge J. J. Snow J. W. Kim J. Orkin S. H. (2009). DNA methyltransferase 1 is essential for and uniquely regulates hematopoietic stem and progenitor cells. Cell Stem Cell 5, 5442–5449. 10.1016/j.stem.2009.08.016 PMC2767228 19796624

[B219] Tu F. Pang Q. Huang T. Zhao Y. Liu M. Chen X. (2017). Apigenin ameliorates post-stroke cognitive deficits in rats through histone acetylation-mediated neurochemical alterations. Med. Sci. Monit. 23, 234004–234013. 10.12659/msm.902770 PMC5572783 28821706

[B220] Urias B. S. Pavan A. R. Albuquerque G. R. Prokopczyk I. M. Alves T. M. F. de Melo T. R. F. (2022). Optimization of resveratrol used as a scaffold to design histone deacetylase (HDAC-1 and HDAC-2) inhibitors. Pharm. (Basel) 15, 1260. 10.3390/ph15101260 PMC9611521 36297372

[B221] Vaquero A. Scher M. B. Lee D. H. Sutton A. Cheng H. L. Alt F. W. (2006). SirT2 is a histone deacetylase with preference for histone H4 Lys 16 during mitosis. Genes Dev. 20, 201256–201261. 10.1101/gad.1412706 PMC1472900 16648462

[B222] Verma R. Ritzel R. M. Crapser J. Friedler B. D. McCullough L. D. (2019). Evaluation of the neuroprotective effect of Sirt3 in experimental stroke. Transl. Stroke Res. 10, 1057–1066. 10.1007/s12975-017-0603-x PMC9527822 29302794

[B223] Wan D. Zhou Y. Wang K. Hou Y. Hou R. Ye X. (2016). Resveratrol provides neuroprotection by inhibiting phosphodiesterases and regulating the cAMP/AMPK/SIRT1 pathway after stroke in rats. Brain Res. Bull. 121, 121255–121262. 10.1016/j.brainresbull.2016.02.011 26876758

[B224] Wang B. Zhang Y. Cao W. Wei X. Chen J. Ying W. (2016). SIRT2 plays significant roles in lipopolysaccharides-induced neuroinflammation and brain injury in mice. Neurochem. Res. 41, 412490–412500. 10.1007/s11064-016-1981-2 27350577

[B225] Wang H. K. Su Y. T. Ho Y. C. Lee Y. K. Chu T. H. Chen K. T. (2023). HDAC1 is involved in neuroinflammation and blood-brain barrier damage in stroke pathogenesis. J. Inflamm. Res. 16, 164103–164116. 10.2147/jir.S416239 PMC10516226 37745794

[B226] Wang J. Tan S. Zhang Y. Xu J. Li Y. Cheng Q. (2024). Set7/9 aggravates ischemic brain injury via enhancing glutamine metabolism in a blocking Sirt5 manner. Cell Death Differ. 31, 31511–31523. 10.1038/s41418-024-01264-y PMC11043079 38365969

[B227] Wang J. Zhao H. Fan Z. Li G. Ma Q. Tao Z. (2017). Long noncoding RNA H19 promotes neuroinflammation in ischemic stroke by driving histone deacetylase 1-dependent M1 microglial polarization. Stroke 48, 482211–482221. 10.1161/strokeaha.117.017387 28630232

[B228] Wang L. Dai M. Ge Y. Chen J. Wang C. Yao C. (2022). EGCG protects the mouse brain against cerebral ischemia/reperfusion injury by suppressing autophagy via the AKT/AMPK/mTOR phosphorylation pathway. Front. Pharmacol. 13, 13921394. 10.3389/fphar.2022.921394 PMC9489224 36147330

[B229] Wang X. Peng A. Huang C. (2024). Suppression of colon cancer growth by berberine mediated by the intestinal microbiota and the suppression of DNA methyltransferases (DNMTs). Mol. Cell Biochem. 479, 4792131–4792141. 10.1007/s11010-023-04836-7 37639199

[B230] Wang Z. Leng Y. Tsai L. K. Leeds P. Chuang D. M. (2011). Valproic acid attenuates blood-brain barrier disruption in a rat model of transient focal cerebral ischemia: the roles of HDAC and MMP-9 inhibition. J. Cereb. Blood Flow. Metab. 31, 3152–3157. 10.1038/jcbfm.2010.195 PMC3049473 20978517

[B231] Wang Z. Leng Y. Wang J. Liao H. M. Bergman J. Leeds P. (2016a). Tubastatin A, an HDAC6 inhibitor, alleviates stroke-induced brain infarction and functional deficits: potential roles of α-tubulin acetylation and FGF-21 up-regulation. Sci. Rep. 6, 619626. 10.1038/srep19626 PMC4726180 26790818

[B232] Wang Z. Liu Y. Xue Y. Hu H. Ye J. Li X. (2016b). Berberine acts as a putative epigenetic modulator by affecting the histone code. Toxicol Vitro 36, 3610–3617. 10.1016/j.tiv.2016.06.004 27311644

[B233] Wei X. Yu L. Zhang Y. Li X. Wu H. Jiang J. (2021). The role of tet2-mediated hydroxymethylation in poststroke depression. Neuroscience 461, 461118–461129. 10.1016/j.neuroscience.2021.02.033 33689862

[B234] Williams K. Christensen J. Pedersen M. T. Johansen J. V. Cloos P. A. Rappsilber J. (2011). TET1 and hydroxymethylcytosine in transcription and DNA methylation fidelity. Nature 473, 473343–473348. 10.1038/nature10066 PMC3408592 21490601

[B235] Wu D. Lu W. Wei Z. Xu M. Liu X. (2018). Neuroprotective effect of sirt2-specific inhibitor AK-7 against acute cerebral ischemia is P38 activation-dependent in mice. Neuroscience 374, 37461–37469. 10.1016/j.neuroscience.2018.01.040 29382550

[B236] Wu H. D'Alessio A. C. Ito S. Xia K. Wang Z. Cui K. (2011). Dual functions of Tet1 in transcriptional regulation in mouse embryonic stem cells. Nature, 473389–473393. 10.1038/nature09934 PMC3539771 21451524

[B237] Wu Q. J. Zhang T. N. Chen H. H. Yu X. F. Lv J. L. Liu Y. Y. (2022). The sirtuin family in health and disease. Signal Transduct. Target Ther. 7402, 402. 10.1038/s41392-022-01257-8 PMC9797940 36581622

[B238] Wu Z. Zhang Y. Zhang Y. Zhao P. (2020). Sirtuin 2 inhibition attenuates sevoflurane-induced learning and memory deficits in developing rats via modulating microglial activation. Cell Mol. Neurobiol. 40, 40437–40446. 10.1007/s10571-019-00746-9 PMC11449016 31713761

[B239] Xia Q. Gao S. Han T. Mao M. Zhan G. Wang Y. (2022). Sirtuin 5 aggravates microglia-induced neuroinflammation following ischaemic stroke by modulating the desuccinylation of Annexin-A1. J. Neuroinflammation 19301, 301. 10.1186/s12974-022-02665-x PMC9753274 36517900

[B240] Xia Q. Yu Y. Zhan G. Zhang X. Gao S. Han T. (2024). The sirtuin 5 inhibitor MC3482 ameliorates microglia-induced neuroinflammation following ischaemic stroke by upregulating the succinylation level of annexin-A1. J. Neuroimmune Pharmacol. 19, 1917. 10.1007/s11481-024-10117-x 38717643

[B241] Xia Y. Wang J. Liu T. J. Yung W. K. Hunter T. Lu Z. (2007). c-Jun downregulation by HDAC3-dependent transcriptional repression promotes osmotic stress-induced cell apoptosis. Mol. Cell 25, 25219–25232. 10.1016/j.molcel.2007.01.005 PMC1829326 17244530

[B242] Xiao R. Wang Q. Peng J. Yu Z. Zhang J. Xia Y. (2023). BMSC-derived exosomal Egr2 ameliorates ischemic stroke by directly upregulating SIRT6 to suppress Notch signaling. Mol. Neurobiol. 60, 601–617. 10.1007/s12035-022-03037-5 36208355

[B243] Xiaowei X. Qian X. Dingzhou Z. (2023). Sirtuin-3 activates the mitochondrial unfolded protein response and reduces cerebral ischemia/reperfusion injury. Int. J. Biol. Sci. 19, 194327–194339. 10.7150/ijbs.86614 PMC10496505 37705748

[B244] Xin Y. J. Yuan B. Yu B. Wang Y. Q. Wu J. J. Zhou W. H. (2015). Tet1-mediated DNA demethylation regulates neuronal cell death induced by oxidative stress. Sci. Rep. 57645, 7645. 10.1038/srep07645 PMC4284502 25561289

[B245] Xiong X. Y. Pan X. R. Luo X. X. Wang Y. F. Zhang X. X. Yang S. H. (2024). Astrocyte-derived lactate aggravates brain injury of ischemic stroke in mice by promoting the formation of protein lactylation. Theranostics 14, 144297–144317. 10.7150/thno.96375 PMC11303085 39113798

[B246] Xu C. Fu X. Qin H. Yao K. (2024). Traversing the epigenetic landscape: DNA methylation from retina to brain in development and disease. Front. Cell Neurosci. 18, 181499719. 10.3389/fncel.2024.1499719 PMC11637887 39678047

[B247] Yamaguchi S. Hong K. Liu R. Inoue A. Shen L. Zhang K. (2013). Dynamics of 5-methylcytosine and 5-hydroxymethylcytosine during germ cell reprogramming. Cell Res. 23, 23329–23339. 10.1038/cr.2013.22 PMC3587712 23399596

[B248] Yamaguchi S. Hong K. Liu R. Shen L. Inoue A. Diep D. (2012). Tet1 controls meiosis by regulating meiotic gene expression. Nature 492, 492443–492447. 10.1038/nature11709 PMC3528851 23151479

[B249] Yang C. S. Fang M. Lambert J. D. Yan P. Huang T. H. (2008). Reversal of hypermethylation and reactivation of genes by dietary polyphenolic compounds. Nutr. Rev. 66 (Suppl. 1S18-20), S18–S20. 10.1111/j.1753-4887.2008.00059.x 18673481 PMC2829855

[B250] Yang J. Tian X. Yang J. Cui J. Jiang S. Shi R. (2017). 5-Aza-2'-deoxycytidine, a DNA methylation inhibitor, induces cytotoxicity, cell cycle dynamics and alters expression of DNA methyltransferase 1 and 3A in mouse hippocampus-derived neuronal HT22 cells. J. Toxicol. Environ. Health A 80, 801222–801229. 10.1080/15287394.2017.1367143 28880816

[B251] Yang Q. Chen L. Zhang H. Li M. Sun L. Wu X. (2024). DNMT1 regulates human erythropoiesis by modulating cell cycle and endoplasmic reticulum stress in a stage-specific manner. Cell Death Differ. 31, 31999–41012. 10.1038/s41418-024-01305-6 PMC11303534 38719927

[B252] Yang R. Shen Y. J. Chen M. Zhao J. Y. Chen S. H. Zhang W. (2022). Quercetin attenuates ischemia reperfusion injury by protecting the blood-brain barrier through Sirt1 in MCAO rats. J. Asian Nat. Prod. Res. 24, 24278–24289. 10.1080/10286020.2021.1949302 34292112

[B253] Yang X. Geng K. Zhang J. Zhang Y. Shao J. Xia W. (2017). Sirt3 mediates the inhibitory effect of adjudin on astrocyte activation and glial scar formation following ischemic stroke. Front. Pharmacol. 8943, 943. 10.3389/fphar.2017.00943 PMC5744009 29311941

[B254] Yildirim F. Ji S. Kronenberg G. Barco A. Olivares R. Benito E. (2014). Histone acetylation and CREB binding protein are required for neuronal resistance against ischemic injury. PLoS One 9, 9e95465. 10.1371/journal.pone.0095465 PMC3991684 24748101

[B255] Yin J. Han P. Tang Z. Liu Q. Shi J. (2015). Sirtuin 3 mediates neuroprotection of ketones against ischemic stroke. J. Cereb. Blood Flow. Metab. 35, 351783–351789. 10.1038/jcbfm.2015.123 PMC4635233 26058697

[B256] Yin R. Mao S. Q. Zhao B. Chong Z. Yang Y. Zhao C. (2013). Ascorbic acid enhances Tet-mediated 5-methylcytosine oxidation and promotes DNA demethylation in mammals. J. Am. Chem. Soc. 135, 13510396–13510403. 10.1021/ja4028346 23768208

[B257] Yu H. Su Y. Shin J. Zhong C. Guo J. U. Weng Y. L. (2015). Tet3 regulates synaptic transmission and homeostatic plasticity via DNA oxidation and repair. Nat. Neurosci. 18, 18836–18843. 10.1038/nn.4008 PMC4446239 25915473

[B258] Yuan H. Denton K. Liu L. Li X. J. Benashski S. McCullough L. (2016). Nuclear translocation of histone deacetylase 4 induces neuronal death in stroke. Neurobiol. Dis. 91, 91182–91193. 10.1016/j.nbd.2016.03.004 26969532

[B259] Yuan K. Wu Q. Yao Y. Shao J. Zhu S. Yang J. (2024). Deacetylase SIRT2 inhibition promotes microglial M2 polarization through axl/PI3K/AKT to alleviate white matter injury after subarachnoid hemorrhage. Transl. Stroke Res. 10.1007/s12975-024-01282-5 39103659

[B260] Zeng X. He G. Yang X. Xu G. Tang Y. Li H. (2022). Zebularine protects against blood-brain-barrier (BBB) disruption through increasing the expression of zona occludens-1 (ZO-1) and vascular endothelial (VE)-cadherin. Bioengineered 13, 134441–134454. 10.1080/21655979.2021.2024323 PMC8974047 35112992

[B261] Zhang L. He X. Liu L. Jiang M. Zhao C. Wang H. (2016). Hdac3 interaction with p300 histone acetyltransferase regulates the oligodendrocyte and astrocyte lineage fate switch. Dev. Cell 36, 36316–36330. 10.1016/j.devcel.2016.01.002 PMC4750051 26859354

[B262] Zhang L. Y. Zhang S. Y. Wen R. Zhang T. N. Yang N. (2024). Role of histone deacetylases and their inhibitors in neurological diseases. Pharmacol. Res. 208, 208107410. 10.1016/j.phrs.2024.107410 39276955

[B263] Zhang M. Wang J. Li J. Kong F. Lin S. (2023). miR-101-3p improves neuronal morphology and attenuates neuronal apoptosis in ischemic stroke in young mice by downregulating HDAC9. Transl. Neurosci. 14, 1420220286. 10.1515/tnsci-2022-0286 PMC10224617 37250142

[B264] Zhang Q. Li D. Zhao H. Zhang X. (2022). Decitabine attenuates ischemic stroke by reducing astrocytes proliferation in rats. PLoS One 17, 17e0272482. 10.1371/journal.pone.0272482 PMC9345475 35917376

[B265] Zhang R. R. Cui Q. Y. Murai K. Lim Y. C. Smith Z. D. Jin S. (2013). Tet1 regulates adult hippocampal neurogenesis and cognition. Cell Stem Cell 13, 13237–13245. 10.1016/j.stem.2013.05.006 PMC4474382 23770080

[B266] Zhang Y. Anoopkumar-Dukie S. Mallik S. B. Davey A. K. (2021). SIRT1 and SIRT2 modulators reduce LPS-induced inflammation in HAPI microglial cells and protect SH-SY5Y neuronal cells *in vitro* . J. Neural Transm. (Vienna) 128, 128631–128644. 10.1007/s00702-021-02331-1 33821324

[B267] Zhang Y. Li Y. Li J. Li B. Chong Y. Zheng G. (2019a). SIRT1 alleviates isoniazid-induced hepatocyte injury by reducing histone acetylation in the IL-6 promoter region. Int. Immunopharmacol. 67, 67348–67355. 10.1016/j.intimp.2018.11.054 30578970

[B268] Zhang Y. Zhang Y. Chen D. Wang C. Chen L. Gao C. (2019b). Genome-wide alteration of 5-hydroxymethylcytosine in hypoxic-ischemic neonatal rat model of cerebral palsy. Front. Mol. Neurosci. 12214, 214. 10.3389/fnmol.2019.00214 PMC6737274 31551709

[B269] Zhao A. Xu W. Han R. Wei J. Yu Q. Wang M. (2024). Role of histone modifications in neurogenesis and neurodegenerative disease development. Ageing Res. Rev. 98, 98102324. 10.1016/j.arr.2024.102324 38762100

[B270] Zhao X. Benveniste E. N. (2008). Transcriptional activation of human matrix metalloproteinase-9 gene expression by multiple co-activators. J. Mol. Biol. 383945-956, 945–956. 10.1016/j.jmb.2008.08.071 PMC2748421 18790699

[B271] Zhao Y. Mu H. Huang Y. Li S. Wang Y. Stetler R. A. (2022). Microglia-specific deletion of histone deacetylase 3 promotes inflammation resolution, white matter integrity, and functional recovery in a mouse model of traumatic brain injury. J. Neuroinflammation 19201, 201. 10.1186/s12974-022-02563-2 PMC9357327 35933343

[B272] Zhong L. Yan J. Li H. Meng L. (2020). HDAC9 silencing exerts neuroprotection against ischemic brain injury via miR-20a-dependent downregulation of NeuroD1. Front. Cell Neurosci. 14, 14544285. 10.3389/fncel.2020.544285 PMC7873949 33584204

[B273] Zhou D. Huang Z. Zhu X. Hong T. Zhao Y. (2021). Circular RNA 0025984 ameliorates ischemic stroke injury and protects astrocytes through miR-143-3p/TET1/ORP150 pathway. Mol. Neurobiol. 58, 585937–585953. 10.1007/s12035-021-02486-8 34435328

[B274] Zhou Y. Li C. Wu R. Yin H. Liu G. Meng H. (2024). Molecular imaging reveals antineuroinflammatory effects of HDAC6 inhibition in stroke models. Mol. Pharm. 21, 216433–216443. 10.1021/acs.molpharmaceut.4c01006 39504500

[B275] Zhou Z. Xu N. Matei N. McBride D.W. Ding Y. Liang H. (2021). Sodium butyrate attenuated neuronal apoptosis via GPR41/Gβγ/PI3K/Akt pathway after MCAO in rats. J. Cereb. Blood Flow. Metab. 41, 41267–41281. 10.1177/0271678x20910533 PMC8370004 32151222

[B276] Zhu S. Zhang Z. Jia L. Q. Zhan K. X. Wang L. J. Song N. (2019). Valproic acid attenuates global cerebral ischemia/reperfusion injury in gerbils via anti-pyroptosis pathways. Neurochem. Int. 124, 124141–124151. 10.1016/j.neuint.2019.01.003 30611759

[B277] Zhu X. Girardo D. Govek E. E. John K. Mellén M. Tamayo P. (2016). Role of tet1/3 genes and chromatin remodeling genes in cerebellar circuit formation. Neuron 89, 89100–89112. 10.1016/j.neuron.2015.11.030 PMC4707072 26711116

[B278] Zhu X. Liu Q. Zhu F. Jiang R. Lu Z. Wang C. (2024). An engineered cellular carrier delivers miR-138-5p to enhance mitophagy and protect hypoxic-injured neurons via the DNMT3A/Rhebl1 axis. Acta Biomater. 186, 186424–186438. 10.1016/j.actbio.2024.07.059 39122135

